# Short-term consumption of the modified standard American diet perturbed the metabolic balance and altered DNA damage in MMTV-PyMT transgenic mice

**DOI:** 10.1186/s13058-025-02075-w

**Published:** 2025-07-25

**Authors:** Arlet Hernandez, Alekhya Puppala, Jenna Hedlich-Dwyer, Nayonika Mukherjee, Guihua Zhai, Valeria L. Dal Zotto, Bohan Ning, Hua Guo, Ritu Aneja, Natalie R. Gassman

**Affiliations:** 1 Department of Pharmacology and Toxicology, Heersink School of Medicine, The University of Alabama at Birmingham, 1720 University Blvd, Birmingham, AL 35294 USA; 2 Department of Nutrition Sciences, School of Health Professions, The University of Alabama at Birmingham, 1716 9th Ave South, Birmingham, AL 35294 USA; 3 Center for Clinical and Translational Sciences, Biostatistics, Epidemiology & Research Design, The University of Alabama at Birmingham, 1825 University Blvd, Birmingham, AL 35294 USA; 4 Department of Pathology, Heersink School of Medicine, The University of Alabama at Birmingham, 1802 6th Ave South, Birmingham, AL 35233 USA

**Keywords:** Breast cancer, American diet, Adiposity, Oxidative stress, DNA damage, Foxm1

## Abstract

**Background:**

Risk factors for breast cancer include obesity and hyperglycemia, which are associated with poor survival. Previous studies have used high-fat diets (HFDs) or Western-style diets to model dietary influences on breast cancer progression. However, these diets do not reflect the energy-dense, nutrient-poor diets that Americans typically consume. To address this gap in our understanding of the interplay between diet and breast cancer progression, we examined the effects of a modified standard American diet (SAD2) on mammary tumorigenesis in the MMTV-PyMT transgenic murine model and their FVB/N controls.

**Methods:**

MMTV-PyMT and FVB/N mice were fed normal chow or the experimental diet SAD2 for up to 12 weeks. We evaluated body weight, blood glucose, adiposity, cytokine, and tumor characteristics to measure SAD2 diet-induced changes in breast tumor development.

**Results:**

Increased body weight and adiposity were observed in MMTV SAD2-treated mice, consistent with the findings of shorter-term HFD studies. The SAD2 diet also resulted in earlier tumor initiation and progression and decreased survival in the SAD2-fed mice. While only modest changes were observed in circulating cytokines and metabolic parameters, the SAD2 tumors presented significant changes in oxidative DNA damage and advanced glycation end products (AGEs). These changes coincided with increases in the oncogenic transcription factor Foxm1 and the expression of Glut1. Both proteins are elevated in breast cancer patient samples but have not yet been linked to diet-induced effects.

**Conclusions:**

Using SAD2, we demonstrated that an American-style diet increased weight and adiposity while promoting the accumulation of oxidative DNA damage and AGEs and the expression of oncogenic Foxm1 within a relatively short diet interval. These data suggest that the SAD2 diet may offer insight into mechanisms that promote breast cancer aggressiveness and resistance to therapy.

**Supplementary Information:**

The online version contains supplementary material available at 10.1186/s13058-025-02075-w.

## Background

Breast cancer is the most commonly diagnosed cancer in women and the second leading cause of death in the United States and worldwide [1]. In 2024, an estimated 310,720 new cases of invasive breast cancer are projected to be diagnosed in US women; an estimated 42,250 women will succumb to breast cancer [2]. Risk factors for breast cancer include obesity and hyperglycemia, the incidence of which has been growing rapidly over the last few decades [3]. In the US, more than two-thirds of adults are considered overweight, with a body mass index (BMI) of 25–29.9, or obese, with a BMI > 30. A meta-analysis of studies involving patients with breast cancer revealed that the risk of recurrence or death was 30% greater in women with obesity than in those without obesity [4]. Obesity is recognized as a risk factor for post-menopausal breast cancer and is associated with poor survival, regardless of menopausal status [5, 6]. Moreover, the risk of recurrence and death among patients with early-stage breast cancer is greater in women with obesity than in those without [7, 8]. The relationships between obesity and the molecular subtypes of breast cancer remain unknown [9–11]. However, high body mass is associated with poor outcomes for patients with the luminal/human epidermal growth factor 2 (HER)-positive and triple-negative subtypes [12, 13].

Given the poor understanding of the associations between obesity and breast cancer, there is significant interest in understanding the molecular mechanisms that contribute to increased aggressiveness, metastasis, and poor survival in obese women. Preclinical models frequently use diet-induced obesity, and there are also single-gene mutation models [14]. High-fat diets (HFDs, ~ 60% fat) or Western-style diets (40–50% fat) are also frequently used to increase body mass and adipose tissue in mice [14–19]. Studies using these models have established enhanced tumorigenesis and metastatic potential in luminal and triple-negative subtypes [15–19]. The levels of inflammatory cytokines, such as leptin, monocyte chemotactic protein-1 (MCP-1), resistin, and tumor necrosis factor-α (TNFα), are also elevated under HFD conditions [15, 16]. However, these dietary models do not reflect the diet that most Americans consume [20]. The increasing obesity rate in the US is attributed to energy-dense, nutrient-poor diets, which feature high mixtures of fats and carbohydrates. Over the past several years, new models have been published that better reflect the standard American diet (SAD), capturing the mixtures of fats and carbohydrates consumed by Americans. Several studies in rats and mice have used the SAD diet and SAD2, a modified version high in carbohydrates and saturated and polyunsaturated fatty acids but lacking trans-fat [20–22]. Long-term consumption of the SAD2 diet results in chronic inflammation, glucose intolerance, and impaired immune function in nontumored animal models [20–22].

The purpose of this study was to examine the effects of the SAD2 diet on mammary tumorigenesis in the MMTV-PyMT transgenic murine model. The MMTV-PyMT mouse is a commonly used genetically engineered mouse model of breast cancer that shows rapid development of multifocal tumors with extensive lung metastases [23]. Tumorigenesis occurs in the luminal cells of the mammary gland and progresses through distinct histological stages, mimicking human ductal breast cancer progression [23]. Tumors develop on a well-characterized timeline, with hyperplasia occurring at 4–6 weeks and late carcinoma occurring at 10–14 weeks [23, 24]. Tumors are negative for estrogen and progesterone receptors, with HER2 being overexpressed during malignant transformation [24]. The tumors are genetically profiled as the luminal B subtype [25]. Using this model, we examined the dietary effects of SAD2 on MMTV-PyMT mice and their FVB/N background controls. We also examined cytokines, metabolic parameters, proliferation, angiogenesis, DNA damage and repair markers to determine their role in tumorigenesis under the SAD2 diet.

## Methods

### Animals

All experiments were approved by the University of Alabama at Birmingham (UAB) Institutional Animal Care and Use Committee (IACUC-22460). Female MMTV-PyMT (mouse mammary tumor virus-polyoma middle tumor-antigen; thereafter MMTV) and FVB/N (thereafter FVB) mice were bred and weaned at 3.5 weeks of age (Jackson Laboratories, Bar Harbor, ME, USA). The mice were genotyped three days after weaning and were allowed access to food and water *ad libitum*. The mice were fed normal chow or the experimental diet SAD2 for up to 12 weeks. The body weights of the mice were measured twice weekly, food intake was recorded weekly for each cage, and nutrient intake parameters were calculated based on these records. Tumor size was measured twice weekly. The animals continued their respective diets for up to 12 weeks or until the tumor volume measured at the biweekly interval was at or exceeded 1500 mm^3^ or a human endpoint was reached. The animals were also observed for distress or pain and were sacrificed when appropriate. The progression of body weight changes was evaluated in the MMTV and FVB mice. The tumor number, tumor volume, and overall survival are only shown for the MMTV mice. The linear mixed effects model was fitted for longitudinal data, and a two-way ANOVA with Tukey’s multiple comparisons as a post hoc test was performed. The significance of terminal body weights was analyzed via the Student’s t-test. All analyses were performed with GraphPad Prism version 9.0, and *p* < 0.05 was considered significant.

### Diet

MMTV and FVB mice were assigned to two groups and fed either normal chow (Teklad 7917, Envigo) or the SAD2 diet (TD.180618, Envigo) for up to 12 weeks (Table 1). Normal chow is a standard reference diet commonly used in rodent research. The SAD2 diet consists of increased fat and carbohydrate contents, comprising white flour, corn starch, and a combination of oils, including soybean, corn, and cottonseed oil.

**Table 1:** Formulation of the normal Chow diet and the SAD2 diet

Normal Chow (Teklad 7917)		SAD2 (TD.180618)	
Ingredient	%	Ingredient	%
Ground corn	21.0	Casein	14.1
Ground whole wheat	35.37	L-Cystine	0.18
Ground whole oats	10.0	Bleached white flour	41.0
Wheat middling	10.0	Corn starch	2.5
Fish meal	9.0	Maltodextrin	6.0
Soybean meal	5.0	Sucrose	12.67
Soybean oil	1.5	Soybean oil	1.73
Alfalfa meal	2.0	Corn oil	1.06
Corn gluten	2.0	Cottonseed oil	2.09
Brewers dried yeast	1.0	Lard	1.80
Dicalcium phosphate	1.5	Beef tallow	1.60
Limestone	1.5	Anhydrous milk fat	2.33
Salt	0.5	Crisco (vegetable shortening)	5.95
Mineral and vitamin premix	0.63	Cholesterol	0.04
		Cellulose	1.90
		Mineral mix	3.50
		Sodium chloride	0.40
		Vitamin mix	1.00
		Choline bitartrate	0.14
		TBHQ, antioxidant	0.003
**Macronutrients**	**% kcal**	**Macronutrients**	**% kcal**
Protein	24.0	Protein	15.4
Fat	14.0	Fat	35.6
Carbohydrate	62.0	Carbohydrate	49.0

### Tissue collection

Several tumors were collected from each of the mammary glands of each mouse, and their relative locations were noted and weighed individually. Adipose depots from the dorsal, gonadal, and visceral fat were collected, weighed individually, and stored at -80 °C. The internal organs (hearts, livers, and lungs) were also collected. Tumors were divided in half, with one half going into RNAlater (Thermo Fisher, Waltham, MA, USA) and the other being fixed for 48 h in 10% neutral buffered formalin (NBF; Thermo Fisher). Organs were also fixed in NBF. Fixed tumors and organs were processed and embedded in paraffin by UAB’s Comparative Pathology Lab. The tissues were sectioned at 5 μm and mounted onto functionalized glass slides.

### Glucose measurements

An initial blood glucose measurement was performed before the mice were fed the diet. Then, fed blood glucose was measured at weeks 1, 4, 8, and 12 to monitor changes and fasting glucose was measured at week 10. Blood samples were obtained from the tail nick using a sterile single-use lancet. Before taking measurements, the fasted mice had food withdrawn from 8 AM to 12 PM. A fasting duration of 4 h was selected based on Carper et al. [26]. Measurements were performed via the Bayer Contour Next Blood Glucose Monitoring System (ADW Diabetes; Pompano Beach, FL, USA). Blood glucose values were graphed via GraphPad Prism and compared using the Student’s *t*-test.

### Blood collection

The FVB and MMTV mice were anesthetized (with 1–3% isoflurane), and a cardiac puncture was performed via a sterile single-use syringe. Blood was collected in two K2E (K_2_EDTA) blood collection microtainer tubes (BD Microtainer Becton Dickinson, Franklin Lakes, NJ, USA), and plasma was collected. A final concentration of 50 µM of the metabolic inhibitor DPPIV (Millipore Sigma Franklin Lakes, NJ, USA) and a 1X protease cocktail were added to one vial of plasma for metabolic analysis. A second vial of plasma was used for cytokine analysis. Plasma from a subset of mice was used for analysis. EVE technologies perform cytokine/chemokine 32-plex and metabolic hormone discovery assays. A cytokine panel was used to measure eotaxin, granulocyte colony-stimulating factor (G-CSF), interferon gamma (IFNγ), interleukin 1 alpha (IL-1α), interleukin 1 beta (IL-1β), interleukin 2 (IL-2), interleukin 3 (IL-3), interleukin 4 (IL-4), interleukin 5 (IL-5), interleukin 6 (IL-6), interleukin 7 (IL-7), interleukin 9 (IL-9), interleukin 10 (IL-10), interleukin 12 beta (IL-12p40), interleukin 12 (IL-12p70), interleukin 13 (IL-13), interleukin 15 (IL-15), interleukin 17 alpha (IL-17 A), interferon gamma-induced protein 10 (IP-10), keratinocyte chemoattractant (KC), leukemia inhibitory factor (LIF), lipopolysaccharide-induced chemokine (LIX), monocyte chemoattractant protein 1 (MCP-1), macrophage colony-stimulating factor (M-CSF), chemokine ligand 9 (MIG), macrophage inflammatory protein 1 alpha (MIP-1α), macrophage inflammatory protein 1 beta (MIP-1β), macrophage inflammatory protein 2 (MIP-2), chemokine ligand 5 (RANTES), tumor necrosis factor alpha (TNF-α), and vascular endothelial growth factor (VEGF- The metabolic panel measured amylin, connecting peptide 2 (C-peptide 2), and total and active levels of glucose-dependent insulinotropic polypeptide (GIP), glucagon-like peptide 1 (GLP-1), ghrelin, glucagon, insulin, leptin, pancreatic polypeptide (PP), peptide tyrosine tyrosine (PYY), resistin, and secretin. Values obtained from EVE technologies were graphed as a heatmap displaying the median values. Graphs displaying individual values are shown as the means ± SEMs in GraphPad Prism, and comparisons were made using the Student’s *t*-test.

### Immunohistochemistry

A mammary tumor from each MMTV mouse was selected for immunohistochemical analysis. The tissue sections were deparaffinized via three changes of xylene for 5 min each. The slides were rehydrated through a series of 100%, 95%, 70%, and 50% ethanol followed by water, each lasting 5 min. Antigen retrieval was performed via freshly prepared citrate buffer (pH 6.0) or ethylenediaminetetraacetic acid (EDTA) buffer (pH 9.0) (Biocare Medical, Pacheco, CA, USA) at 15 psi for 10‒30 min. The slides were subsequently allowed to cool to room temperature (RT) for 30 min and then washed twice in 1X Tris-buffered saline (TBS; VWR Life Sciences, Radnor, PA, USA) with 0.1% Tween-20 (TBST). Endogenous peroxidase was quenched with hydrogen peroxide (Biocare Medical), and the slides were washed twice in 1X TBST and blocked with 2.5% goat serum (Biocare Medical) or 5% goat serum (Sigma‒Aldrich, Burlington, MA, USA) in phosphate-buffered saline (PBS) for 1 h. This step was followed by incubation with a primary antibody (Table 2). All primary antibodies were diluted with Renoir Red Diluent (Biocare Medical). The slides were rinsed in 1X TBST, and secondary antibody staining was performed for 1 h at RT using MACH-2 rabbit HRP (Biocare Medical). After being rinsed three times with 1X TBST, the tissues were exposed to a 3,3’-diaminobenzidine (DAB) Chromogen Kit (Biocare Medical) until a brown color developed (5–8 min). The slides were rinsed in water, and Mayer’s hematoxylin was applied as a counterstain for 30 s to 2 min, adjusted based on the strength of the DAB stain. A longer counterstaining time was used for more intense DAB staining. This was followed by dehydration in alcohol (50%, 70%, 90%, and 100% ethanol), clearance in xylene, and mounting with mounting media.

**Table 2:** Immunohistochemistry conditions for several markers

Markers	Source	Cat#	Dilution	Incubation time	Antigen retrieval solution	Blocking solution
Ki67	Cell Signaling Technology, Danvers, MA, USA	D3B5	1:150	60 min at RT	Citrate Buffer (pH 6.0)	2.5% goat serum
CD31	Cell Signaling Technology	D8V9E	1:50	60 min at RT	Citrate buffer (pH 6.0)	2.5% goat serum
IL6	Sigma‒Aldrich	ZRB1970	1:475	105 min at RT	Citrate buffer (pH 6.0)	2.5% goat serum
Resistin	Bioss, Woburn, MA, USA	Bs-0795R	1:100	Overnight at 4 °C	Citrate buffer (pH 6.0)	5% goat serum
Adiponectin	Sigma‒Aldrich	PRS3551	1:100	Overnight at 4 °C	Citrate buffer (pH 6.0)	5% goat serum
Beta Catenin	Abcam, Cambridge, UK	AB32572	1:100	Overnight at 4 °C	EDTA buffer (pH 9.0)	5% goat serum

### Tumor scoring

All MMTV tissues were stained with hematoxylin and eosin (H&E) by UAB’s Comparative Pathology Lab and evaluated for the presence of tumor necrosis, microvacuolar and macrovacuolar steatosis, fibrosis, and vascular proliferation by a pathologist. Tumor cell necrosis was characterized by the abrupt transition from viable to nonviable tumor cells and was estimated as a percentage for each tumor. The presence of steatosis was assessed via Oil Red O histochemical staining performed by UAB’s Comparative Pathology Lab on fresh tissue sections, and the percentage of total tumor volume was estimated. Microvacuolar steatosis was defined as multiple positive Oil Red O stains present as small vacuoles within the cytoplasm indenting the nuclei of tumor cells visible at 400X magnification (i.e., 40X objective lens and 10X ocular lens) and macrovacuolar steatosis when large droplets were visible at 100X magnification (i.e., 10X objective lens and 10X ocular lens). Fibrosis and vascular proliferation were recorded for each tissue as either present or absent (1 or 0, respectively). Fibrosis was defined as a discrete focus of fibroblastic proliferation within the tumor nests or at the periphery of the tumor. Microvessel density was assessed via a modified version of a method previously reported by Weidner [27]. Vascular proliferation was considered present when at least one area of invasive carcinoma contained at least 20 grouped capillaries and venules. All the slides were evaluated via an Olympus BX43 light microscope. The values were graphed via GraphPad Prism, with the mean ± SEM for percentages and the frequency distribution for ordinal scores. Student’s *t*-test was used to compare values with percentages, and the Mann‒Whitney test was used to compare ordinal scores between groups.

Tumor scoring in MMTV mice was performed via immunohistochemistry by two pathologists on slides stained for adiponectin, the cluster of differentiation 31 (CD31), antigen Kiel 67 (Ki67), resistin, interleukin (IL-6), and beta-catenin (β-catenin). Antibody binding and scoring were optimized using negative control isotype match Ig control and positive control provided by the pathologists within the Department of Pathology. Examples of negative control slides are provided in the Supplemental Figures. The expression scores were evaluated based on the intensity and proportion of positive cells. After the positive and negative controls were reviewed, the percentage of stained cells and the staining intensity were quantified in at least ten high-power fields per section. For resistin and adiponectin, any level of cytoplasmic staining was considered positive. The staining intensity was scored on a 4-point scale from 0 (negative) to 1 (weak), 2 (moderate), or 3 (strong). The degree of staining was scored as follows: 0, no expression; 1, less than 25% positive cells; 2, 26–50% positive cells; and 3, 51–75% positive cells. For CD31 scoring, any level of membranous or cytoplasmic staining was considered positive. Slides were evaluated based on the combined intensity and distribution of cells positive for CD31, with scores ranging from 1 (low) to 2 (intermediate) and 3 (high). The expression of Ki67 was confined to the nuclei of the tumor cells. The Ki67 score represents the percentage of immunostained nuclei among the total number of nuclei in tumor cells, regardless of immunostaining intensity. The Ki67 scores were classified as follows: 1, < 5%; 2, 5–20%; 3, 21–50%; 4, 51–70%; and 5, > 70%. For IL-6 scoring, any level of cytoplasmic staining was considered positive. Staining was categorized based on the proportion of positive cells as follows: 0, no staining; 1, 0–10%; 2, 11–50%; and 3, > 50%. Finally, β-catenin was considered positive and categorized based on any level of membranous, cytoplasmic and/or nuclear staining. The staining intensity was scored via a five-point scale. The staining proportions were classified as follows: 0, 0–10%; 1, 11–25%; 2, 26–50%; 3, 51–75%; and 4, > 75%. All the slides were evaluated via an Olympus BX43 light microscope. The values were graphed via GraphPad Prism and presented as the means ± SEMs for percentages and frequency distributions for ordinal scores. The Mann‒Whitney test was used to compare ordinal scores between groups.

### Immunofluorescence

A subset of MMTV tumor tissues were selected for the measurement of various markers via immunofluorescence. The tissue sections were deparaffinized via three changes of xylene for 5 min each. To rehydrate the tissue, the slides were washed with 100%, 95%, 70%, or 50% ethanol for 3 min each. The last wash was performed in Milli-Q H_2_O for 5 min. Antigen retrieval was performed via antibody signal enhancer (ASE) buffer containing 10 mM glycine in PBS, 1% Triton-X, and 0.05% Tween-20 [28]. The slides were then heated in ASE buffer solution three times for 18 s with 5 min of rest, followed by two rounds of 15 s of heating and 5 min of rest. The slides were then cooled to RT using Milli-Q H_2_O and dried. The slides were blocked with 5% goat serum for 30 min at RT and incubated with primary antibody overnight (ON). The following dilutions were used for each antibody: forkhead box M1 (Foxm1) (1:500, 13147-1-AP, ProteinTech, Rosemont, IL, USA), poly(ADP-ribose) (PAR) (1:200, ab14460, Abcam), DNA polymerase beta (Polβ) (1:200, ab26343, Abcam), apurinic/apyrimidinic endonuclease 1 (Ape1) (1:200, ab189474, Abcam), advanced glycation end products (AGE) (1:500, ab23722, Abcam), methylglyoxal (MG) (1:200, MABN1838, Millipore Sigma, Burlington, MA, USA), tumor suppressor p53 binding protein 1 (53BP1), (1:500, NB100-304, Novus Biologicals, Centennial, CO, USA) and glucose transporter 1 (Glut1) (1:100, ab115730, Abcam). To confirm antibody specificity, we also stained slides with an isotype Ig control and measured the fluorescent staining for the Ig. Ig intensity levels were recorded at 8.22 × 10^3^ and 4.33 × 10^3^ for the 546 and 647 channels. There are also representative examples in the Supplemental Figures. The next day, the slides were washed three times with 1X TBST for 5 min each. The slides were then incubated for 1 h at RT with the secondary antibodies Alexa Fluor 647 (1:400; Thermo Fisher) and Alexa Fluor 488 (1:400; Thermo Fisher). Slides stained with anti-mitochondria clone 113-1 AlexaFluor conjugate 488 (1:250; MAB1273A4, Thermo Fisher) were then incubated for 1 h at RT. Nuclear stain was added for 15 min before the end of the secondary antibody incubation period, and Hoechst solution (1:800, Thermo Fisher) was used. The slides were washed three times with 1X TBST for 5 min, dried, mounted with Prolong Gold (Thermo Fisher), and allowed to dry. Imaging was conducted using Keyence (BZ-X800; Keyence, Osaka, Japan) and a 10X objective (NA 0.45). Nine images were taken over the center of the tumor, and analysis was performed via Nikon Elements software. The fluorescence intensity was measured via a binary threshold over the tissue in each image field, and the sum of the intensities was obtained. The values are reported as the fluorescence intensity ± SEM and were compared using Student’s *t*-test in GraphPad Prism.

### Repair assisted damage detection (RADD)

RADD, an assay that detects DNA lesions via cocktails of repair enzymes, was used to evaluate DNA lesion content and detect strand breaks within a subset of tumor tissues [29]. The enzymes used in the assay remove the DNA lesions and tag them via digoxigenin-labeled dUTP. We used enzymes to measure oxidative lesions (oxRADD), uracil lesions (UDG), and crosslink-type lesions (T4PDG), as previously described [30], in MMTV tumor sections. The tissue slides were deparaffinized via three 5 min washes in xylene. The samples were rehydrated via 3 min washes in 100%, 95%, 70%, and 50% ethanol and then washed in Milli-Q H_2_O for 5 min. Antigen retrieval was performed via ASE buffer as previously described. The slides were incubated for 1 h at 37 °C in a hybridization oven with lesion removal cocktails containing Fapy-DNA glycosylase (FPG), Endonuclease IV (Endo IV), and Endonuclease VIII (Endo VII) for oxRADD; Endo IV and uracil DNA glycosylase (UDG) for UDG RADD; and Endo IV and T4 pyrimidine dimer glycosylase (T4PDG) for T4PDG. The cocktails were prepared in 1X ThermoPol buffer containing bovine serum albumin (BSA; New England Biolabs, Ipswich, MA, USA). The lesion tagging mixture containing the Klenow fragment (3′-5′ exo-) and digoxigenin-labeled dUTP was added on top, and the tissues were incubated for another 1 h at 37 °C. The slides were washed three times with 1X TBST and blocked for 30 min at RT with 5% goat serum. After blocking, the slides were incubated ON with an anti-digoxigenin primary antibody (1:250; ab420, Abcam, UK). The next day, the slides were washed three times with 1X TBST and incubated with the secondary antibody Alexa Fluor 546 (1:400; Thermo Fisher) for 1 h at RT. The sections were then incubated with 4′,6-diamidino-2-phenylindole (DAPI) (1:400; Thermo Fisher) for 15 min before the secondary antibody incubation period. After incubation, the tissues were washed three times for 5 min, mounted with Prolong Gold (Thermo Fisher), and left to dry ON. The tumor tissue slides were imaged via an all-in-one Keyence with a 10X objective (NA 0.45). Analysis was performed using Nikon Elements software. A binary mask was used to determine the fluorescence intensity, and values were graphed as the fluorescence intensity ± SEM and compared using the Student’s *t*-test.

### Gene expression assays

RNA was extracted from tumors using a Direct-zol RNA Miniprep Plus Kit (Zymo Research, Irvine, CA, USA) according to the manufacturer’s protocol. Tissues were homogenized by adding 100 mg of each tumor to a PYREX tissue grinder (Corning Life Sciences, Tewksbury, MA, USA) with 800 µL of TRIzol reagent (Life Technologies, Carlsbad, CA, USA). The homogenized samples were centrifuged, and the supernatant was transferred to a fresh tube. To purify RNA, equal volumes of 95–100% ethanol and the supernatant were added to a Zymo-Spin IIICG column and centrifuged, and the flow-through was discarded. DNase I treatment was performed for 15 min at RT. The samples were subjected to a series of wash steps and then eluted with 50 µL of DNase/RNase-free water. The concentration of purified RNA was determined using a Nanodrop Lite Plus (Thermo Fisher). cDNA was synthesized using the iScript Reverse Transcription Supermix (Bio-Rad, Hercules, CA, USA) according to the manufacturer’s protocol. iScript RT Supermix, RNA template (1 µg), and nuclease-free water were combined in a final volume of 20 µL and incubated in a T100 Thermal Cycler (Bio-Rad). The cycling conditions were 25 °C for 5 min, 46 °C for 20 min, and 95 °C for 1 min. Gene expression was analyzed using TaqMan gene expression master mix and manufactured primers (Thermo Fisher). TaqMan master mix, assay, endogenous control, cDNA template, and nuclease-free water were combined in a final volume of 20 µL per well in a 96-well plate. The plates were run in a CFX Opus 96 Real-Time PCR System (Bio-Rad) for an initial incubation at 50 °C for 2 min, followed by polymerase activation at 95 °C for 10 min, 40 cycles of denaturation at 95 °C for 15 s, and annealing/extension at 60 °C for 60 s. The TaqMan assays used are listed in Table 3. The results were analyzed using the ∆Ct method. GraphPad Prism was used to plot the values as the means ± SEMs, and Student’s *t*-test was used for comparisons between groups.

**Table 3:** Gene expression TaqMan primers

Target Gene	TaqMan Catalog Number
Actb (endogenous control)	Mm02619580_g1
Foxm1	Mm00514924_m1
Polb	Mm00448234_m1
Apex1	Mm00507805_g1
Il6	Mm00446190_m1
Adipoq	Mm04933656_m1
Retn	Mm00445641_m1

## Results

### SAD2-fed mice have increased body weight

**Fig. 1:**
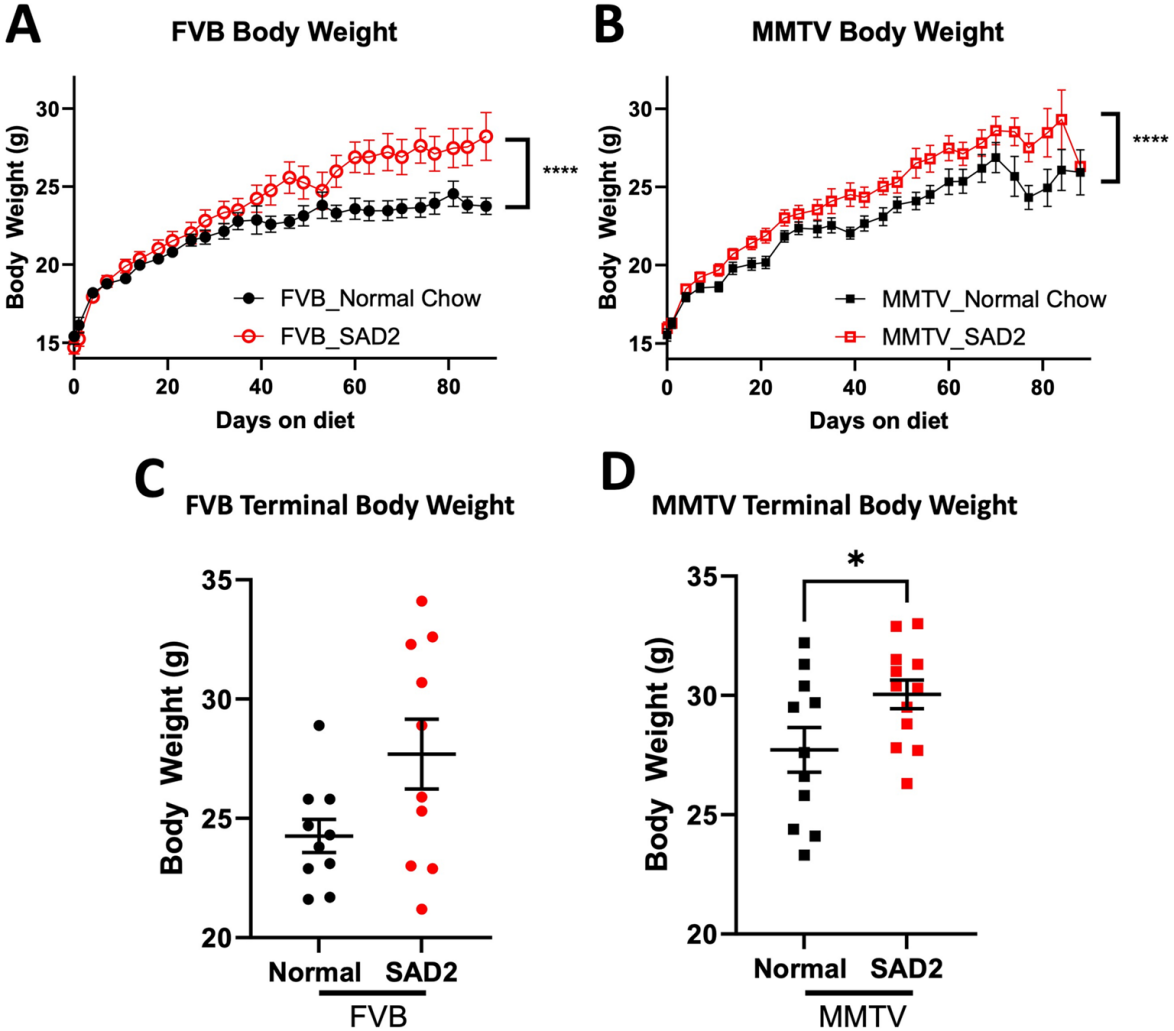
Body weights of FVB and MMTV mice while they consumed the SAD2 diet. The body weights of the FVB and MMTV mice were recorded twice a week for up to 12 weeks on normal chow and SAD2 diets. (**A**) Body weights of the FVB mice throughout the study (*n* = 11 normal chow and *n* = 12 SAD2 at study start). (**B**) Body weights of MMTV mice throughout the study (*n* = 12 normal chow and *n* = 12 SAD2 at study start). (**C**) Body weights of FVB mice at study termination (*n* = 10 normal chow and *n* = 12 SAD2). (**D**) Body weights of MMTV mice before study termination (*n* = 12 normal chow and *n* = 12 SAD2). The graphs are displayed as the means ± SEMs, and significance is displayed as follows: *p < 0.05; ****p < 0.0001

The FVB and MMTV mice were fed normal chow or SAD2 for 12 weeks. Body weight was measured twice weekly throughout the study (Fig. 1). There was a significant increase in the body weight of both FVB and MMTV mice fed the SAD2 diet (Fig. 1A and B). The FVB mice started showing weight divergence on day 40, and the increase in weight persisted until day 88 (Fig. 1A). MMTV mice exhibited increasing body weights in both groups over the study period, which reflects an increasing tumor burden. However, the SAD2-fed MMTV mice had greater body weights than the normal chow-fed MMTV mice from day 18 onward (Fig. 1B). At termination, the body weights of the FVB mice showed the obesity resistance of this background, with ~ 50% of the mice showing weight gain compared with normal chow (Fig. 1C) [31]. For the SAD2 MMTV mice, the body weight at termination was significantly greater in the SAD2-fed group than in the normal chow group (30.04 ± 0.60 vs. 27.72 ± 0.93 g, *p* < 0.05) (Fig. 1D). Diet consumption between the groups was similar throughout the diet period, with the differences in diet reflected in the increased caloric and fat levels within the SAD2 group (Suppl. Fig. 1). Given the observed changes in body weight in the mice fed the SAD2 diet, adipose measurements were evaluated next to understand these changes.

### SAD2 increases adiposity in FVB and MMTV mice

**Fig. 2:**
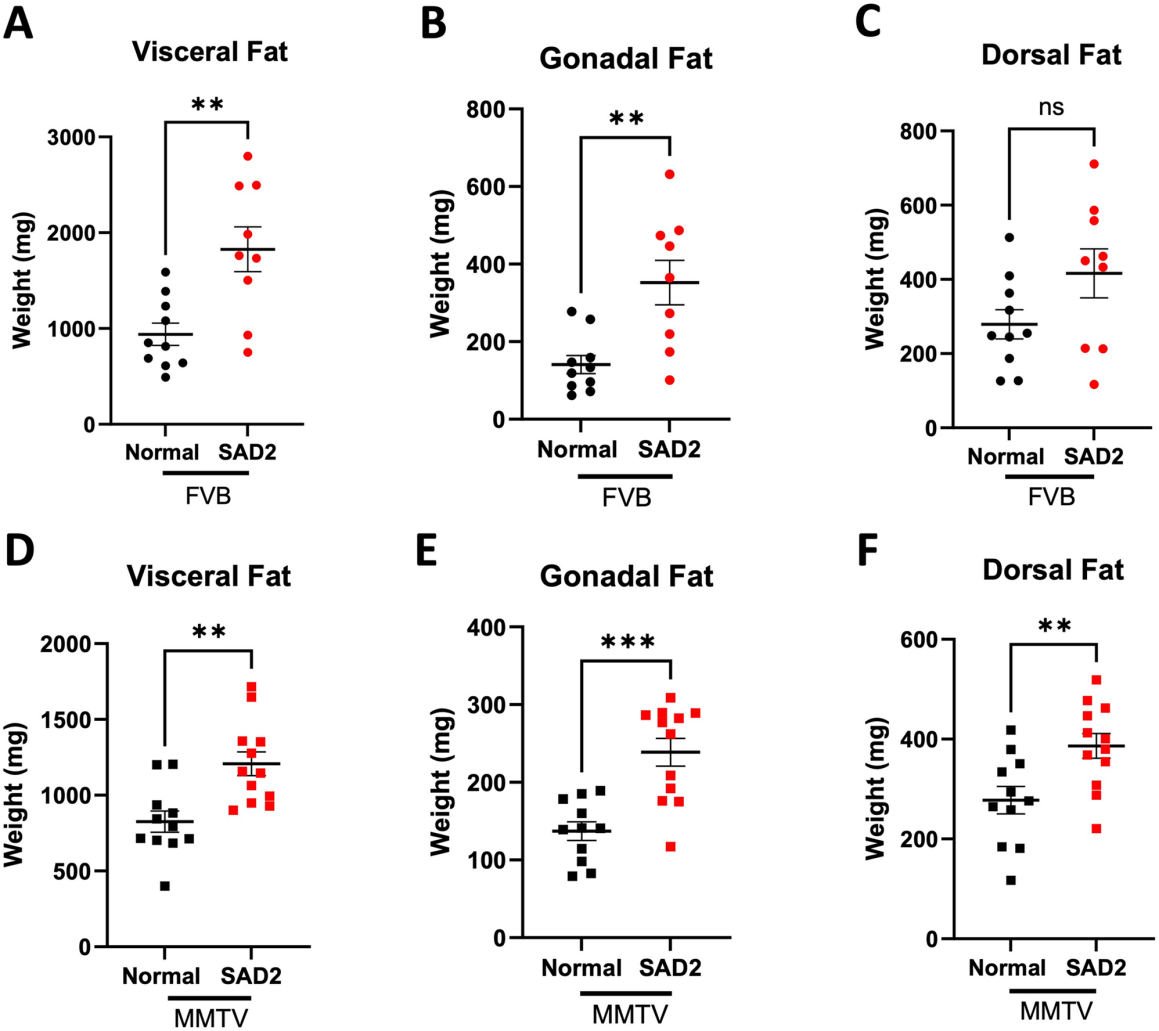
Fat weight in FVB and MMTV mice. Changes in visceral, gonadal, and dorsal fat were measured in the FVB (*n* = 10 normal chow and *n* = 12 SAD2) and MMTV (*n* = 12 normal chow and *n* = 12 SAD2) mice at the termination of the study. (**A**) Visceral fat, (**B**) gonadal fat, and (**C**) dorsal fat in FVB mice. (**D**) Visceral fat, (**E**) gonadal fat, and (**F**) dorsal fat in MMTV mice. The graphs are displayed as the means ± SEMs, with significance as follows: **p < 0.01; ***p < 0.001

Considering the increased body weight of the SAD2-fed mice, we evaluated different types of adipose tissue to better understand these changes in body weight. Visceral, gonadal, and dorsal fat was weighed at termination to assess changes in adipose tissue in FVB and MMTV mice (Fig. 2). Visceral fat is found in the body’s center around the abdominal cavity. Gonadal fat encapsulates the ovaries, and dorsal fat is found between the shoulder blades and includes brown fat [32]. Among the FVB mice, those fed SAD2 had significantly more visceral fat than the normal chow group did (1827 ± 233.5 mg vs. 939.1 ± 116.2 mg, *p* < 0.01) (Fig. 2A). Gonadal fat in the FVB mice was also significantly greater in the SAD2 group than in the normal chow group (352.3 ± 57.8 vs. 140.9 ± 23.4 mg, *p* < 0.01) (Fig. 2B). Differences in dorsal fat between the two diet groups were not significant in the FVB mice (SAD2 416.3 ± 65.8 mg vs. control 279.1 ± 39.1 mg) (Fig. 2C). Among the MMTV mice, visceral fat (1207 ± 78.4 mg vs. 825.2 ± 70.1 mg, *p* < 0.01), gonadal fat (137.1 ± 11.9 mg vs. 238.8 ± 17.8 mg, *p* < 0.001), and dorsal fat (277.9 ± 27.5 mg vs. 386.6 ± 24.7 mg, *p* < 0.01) were significantly greater in the SAD2-fed group than in the normal chow group (Fig. 2D-F).

### SAD2-fed mice have elevated blood glucose levels in FVB and MMTV groups

**Fig. 3:**
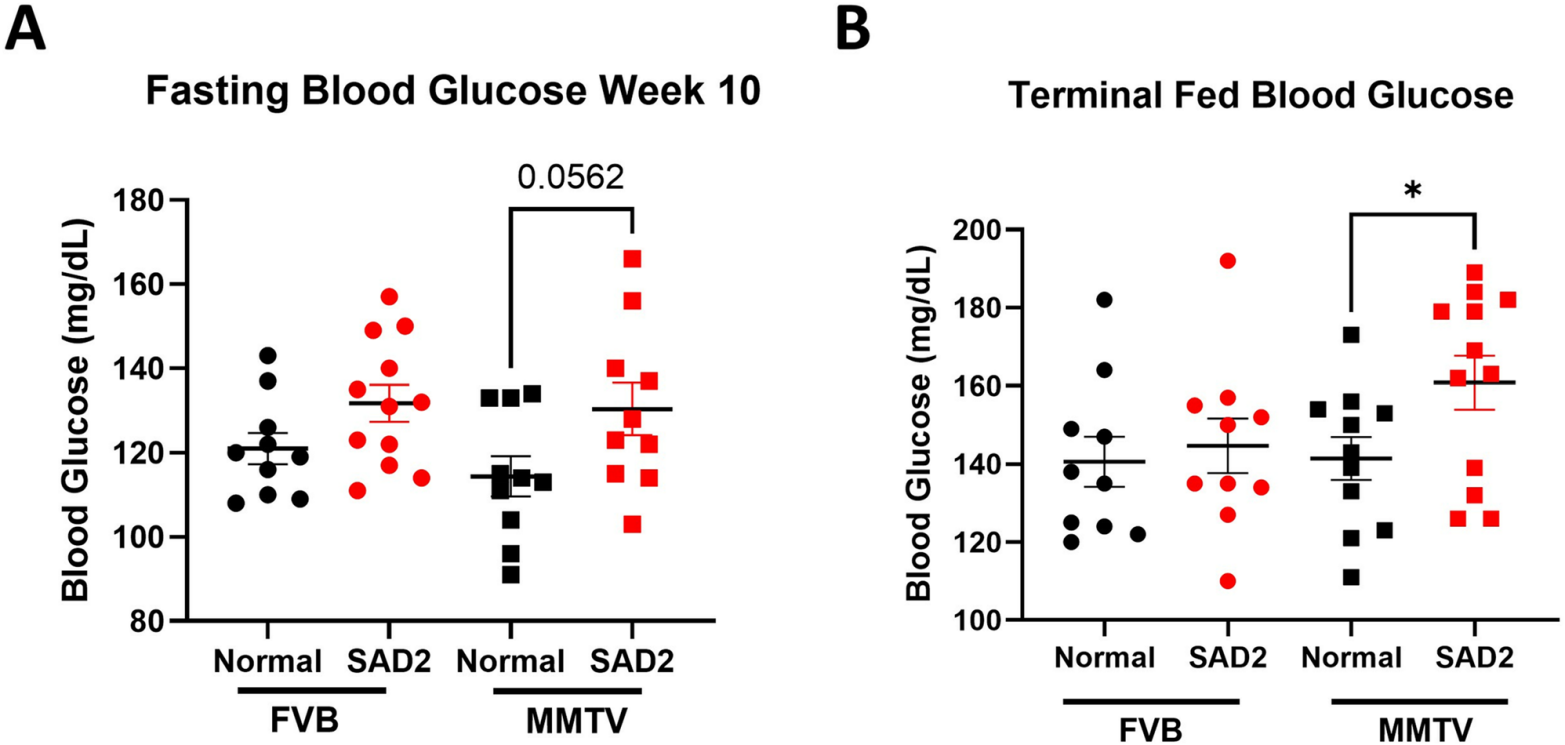
Fasted blood glucose levels in FVB and MMTV mice. (**A**) Fasted glucose measurements were performed at week 10 in FVB (*n* = 10 normal chow and *n* = 12 SAD2) and MMTV (*n* = 10 normal chow and *n* = 10 SAD2) mice. (**B**) Blood glucose measurements were obtained at termination for FVB (*n* = 10 normal chow and *n* = 10 SAD2) and MMTV (*n* = 11 normal chow and *n* = 12 SAD2) mice. Measurements were performed using a Contour Next Blood Glucose Monitoring System. The data are displayed as the means ± SEMs via GraphPad Prism. Significance is displayed as follows: *p < 0.05

Fed blood glucose levels were measured at 1, 4, 8, and 12 weeks to monitor changes in FVB and MMTV mice throughout the study. Fluctuating blood glucose levels were observed in both groups (Suppl. Fig. 2). Fasting blood glucose levels at week 10 in both the FVB and MMTV groups were not significantly greater in the SAD2 group than in the normal chow group (Fig. 3A). Fasting blood glucose levels at week 12 were significantly greater in the SAD2 FVB mice than in the control mice (134.3 ± 3.8 to 121.2 ± 3.9, *p* = 0.03), but too few MMTV mice were observed to have a significant effect (Suppl. Fig. 3). The terminal blood glucose levels were significantly higher in the SAD2 MMTV mice than in control MMTV mice (160.8 ± 6.8 to 141.5 ± 5.5, *p* = 0.04) (Fig. 3B). The SAD2 diet elevated blood glucose levels, although these levels did not reach the threshold indicative of hyperglycemia.

**Fig. 4:**
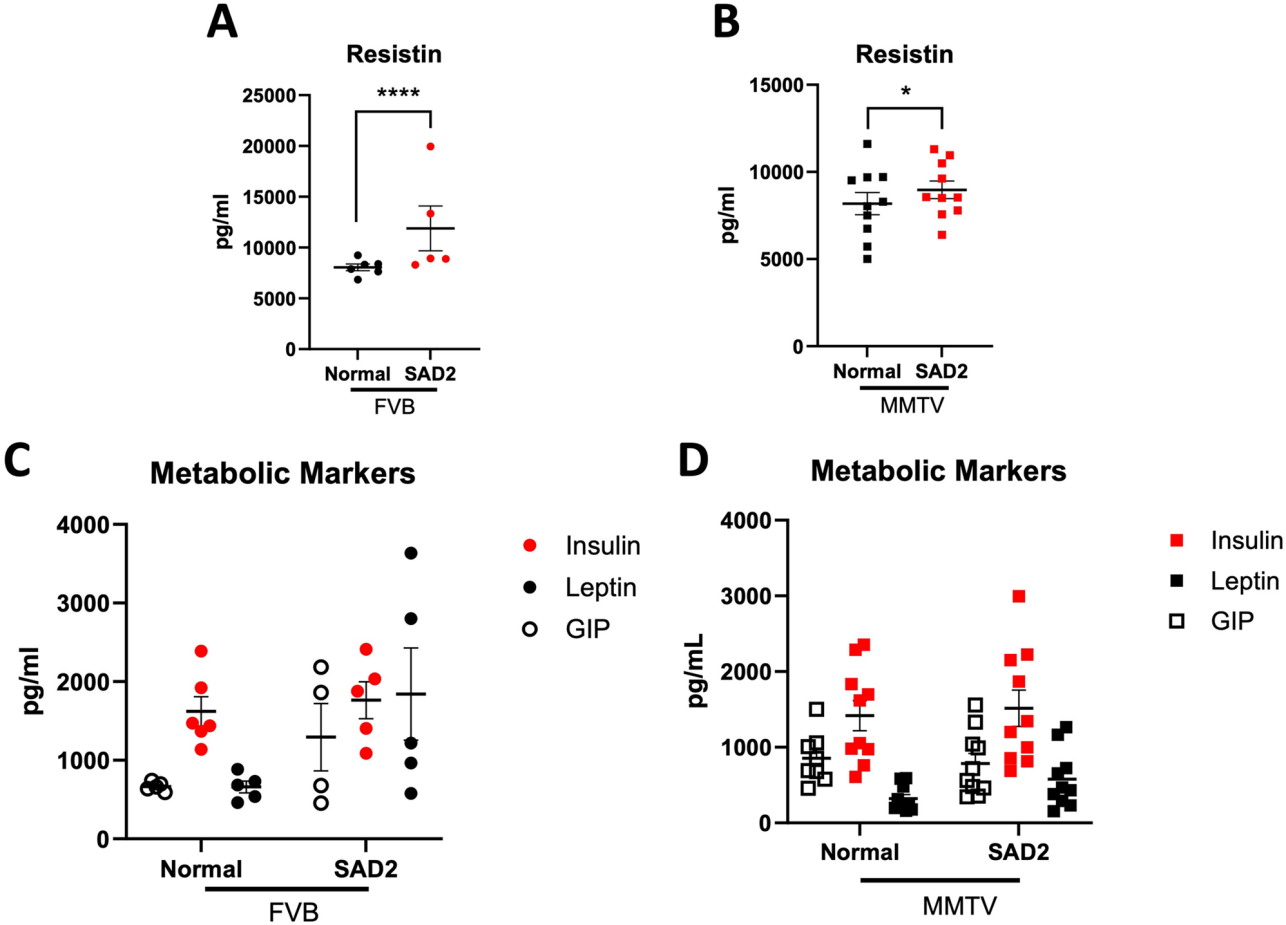
Metabolic markers and hormones in FVB and MMTV mice. Resistin, insulin, leptin, and gastric inhibitory polypeptide (GIP) levels were evaluated in a subset of mice at termination using the EVE technologies metabolic discovery array panel. Resistin levels in (**A**) FVB (*n* = 6 normal and *n* = 5 SAD2) and (**B**) MMTV mice (*n* = 10 normal and *n* = 10 SAD2). Insulin, leptin, and GIP levels in (**C**) FVB (*n* = 6, 5, 5 for normal, respectively and *n* = 6, 5, 4 for SAD2, respectively) and (**D**) MMTV mice (*n* = 10, 10, 8 for normal, respectively and *n* = 10, 10, 10 for SAD2, respectively). GraphPad Prism was used to graph the values as the means ± SEMs, and significance is displayed as follows: *p < 0.05; ****p < 0.0001

As increased blood glucose and adiposity indicate metabolic changes, we next evaluated a panel of metabolic markers using EVE technologies’ metabolic hormone discovery assay array panels using plasma from a subset of FVB and MMTV mice. There were few significant differences within the panel (Suppl. Fig. 4), with only resistin, the hormone secreted from white adipocytes, showing a significant increase in FVB and MMTV mice fed SAD2 compared with those fed normal chow (Fig. 4A and B). We also examined trends in gastric inhibitory polypeptide (GIP), insulin, and leptin levels. GIP stimulates insulin release to regulate blood glucose and nutrient homeostasis. Leptin is a hormone that regulates energy balance by suppressing hunger. Insulin is a peptide hormone that regulates the body’s energy supply by signaling glucose for use. Among FVB mice, nonsignificant increases in GIP and leptin levels were observed in the SAD2 group. Insulin levels did not change significantly with either diet (Fig. 4C). Consistent with the findings from the FVB group, MMTV mice presented no significant differences in insulin levels. However, leptin increased almost twofold, and GIP slightly decreased (Fig. 4D).

**Fig. 5:**
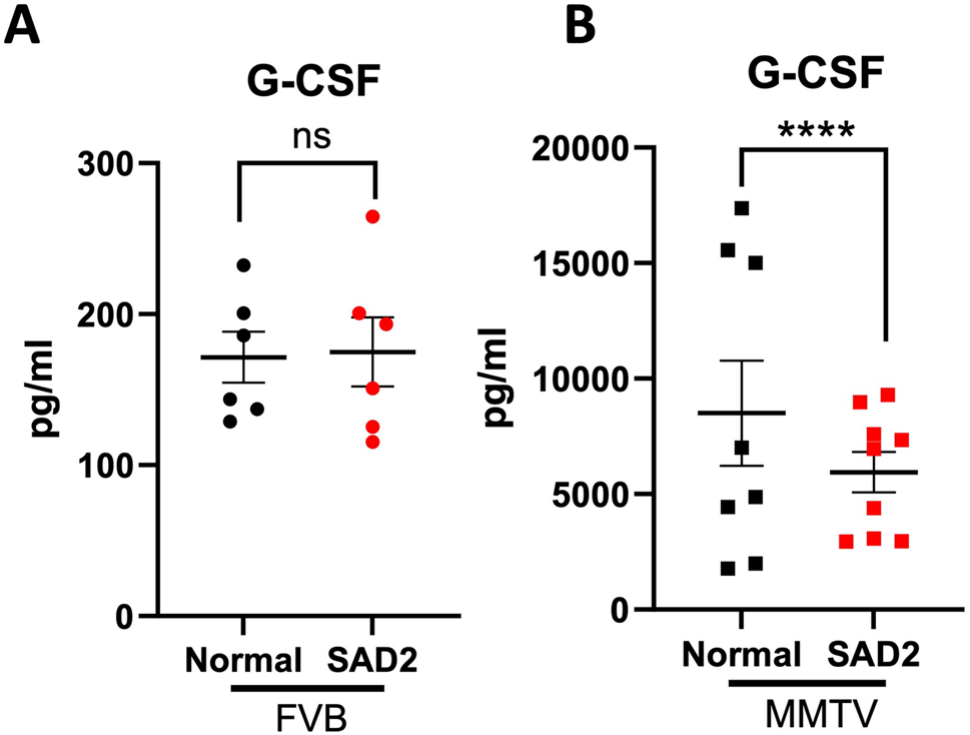
G-CSF levels in FVB and MMTV mice. The cytokine/chemokine 32-plex discovery plex was measured in a subset of mice using EVE technologies, and granulocyte colony-stimulating factor (G-CSF) levels were measured in (**A**) FVB (*n* = 6 per group) and (**B**) MMTV mice (*n* = 8 normal and *n* = 9 SAD2). The values were graphed via GraphPad Prism and are presented as the means ± SEMs. Significance is displayed as follows: ****p < 0.0001

We also screened the plasma for cytokines using EVE technologies’ cytokine/chemokine 32-plex assay to determine whether the diet increased the levels of inflammatory signals. However, the panel exhibited few significant changes (Suppl. Fig. 5). Only granulocyte colony stimulating factor (G-CSF) was significantly lower in the MMTV-SAD2 group than in the normal chow group (Fig. 5). G-CSF is linked to circulating long-chain free fatty acids and energy metabolism.

### SAD2 diet enhances tumor initiation and growth, reducing survival in MMTV mice

**Fig. 6:**
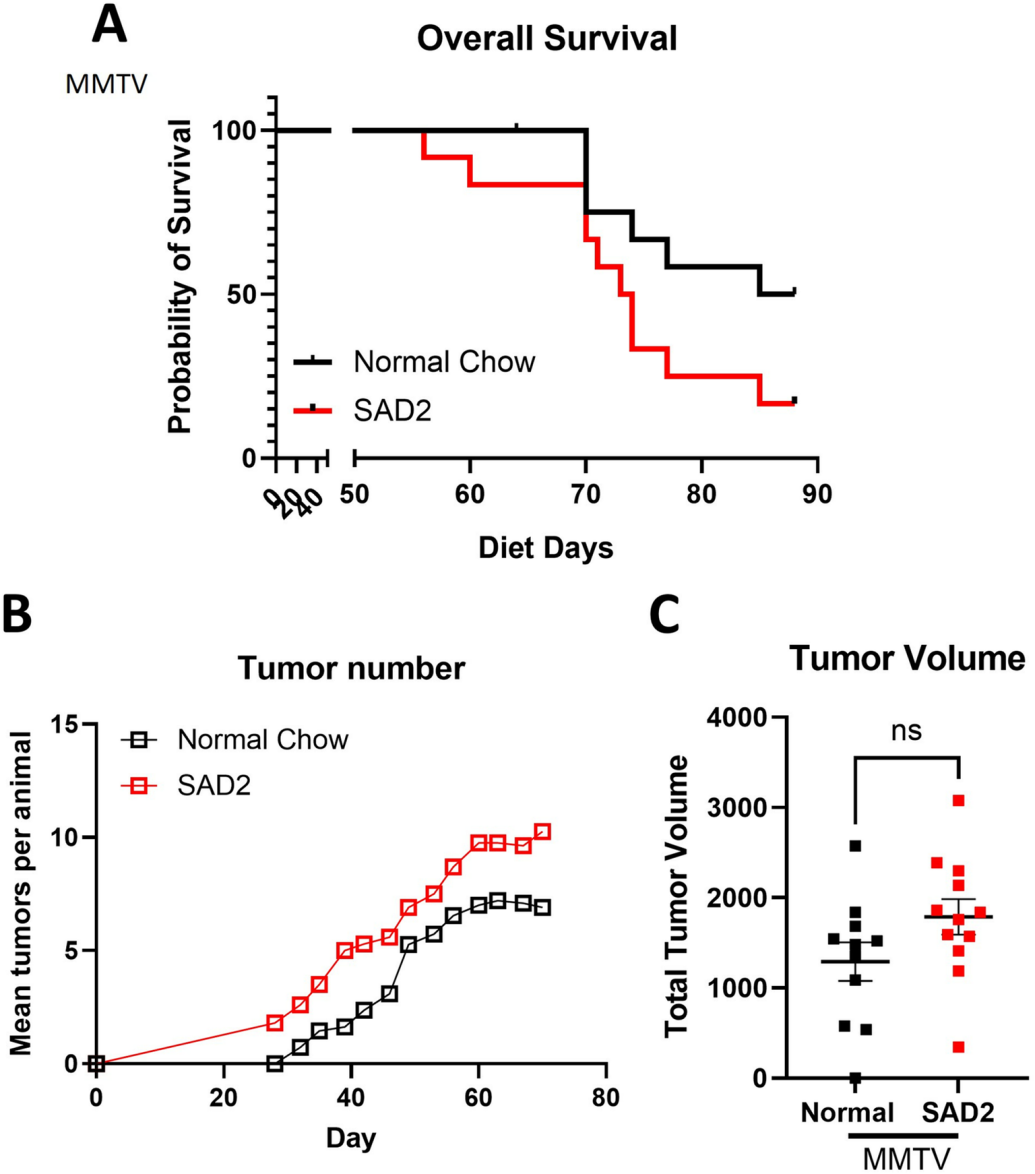
Overall survival and early tumor initiation in SAD2-fed MMTV mice. (**A**) Survival was recorded throughout the study in MMTV mice (*n* = 11 normal chow and *n* = 12 SAD2), and the Kaplan‒Meier method was used to assess the probability of survival after 88 days of diet. (**B**) The mean number of tumors per animal throughout the study period. The tumor numbers and sizes were measured twice per week. The standard deviation in the mean is smaller than the symbols showed, so the error bars cannot be visualized. The standard deviation ranged from day 32 0.08 to 0.24 at day 70. (**C**) Total tumor volume at termination in MMTV mice (*n* = 11 normal chow and *n* = 12 SAD2). The graphs generated via GraphPad Prism are presented as the means ± SEMs

Given the more significant changes in body weight and adiposity, we assessed the effects of SAD2 on tumor characteristics and survival changes during tumor formation in MMTV mice. Tumor size was measured twice weekly. The mice were fed SAD2 for 12 weeks or until their cumulative tumor burden reached 1500 mm^3^. Using these criteria, the median survival of SAD2 MMTV mice was significantly reduced to 73.5 days, in contrast with the 77 days observed in mice fed a normal chow diet (*p* = 0.058) (Fig. 6A). Palpable tumors emerged four days earlier in the mice fed the SAD2 diet than in those fed normal chow. The tumors also exhibited a faster growth rate in the SAD2 group, with the mean numbers per animal increasing more rapidly than the normal chow (Fig. 6B). We also observed that the tumor volume was greater in the SAD2 group than in the normal chow group, although the difference was not statistically significant (Fig. 6C).

**Fig. 7:**
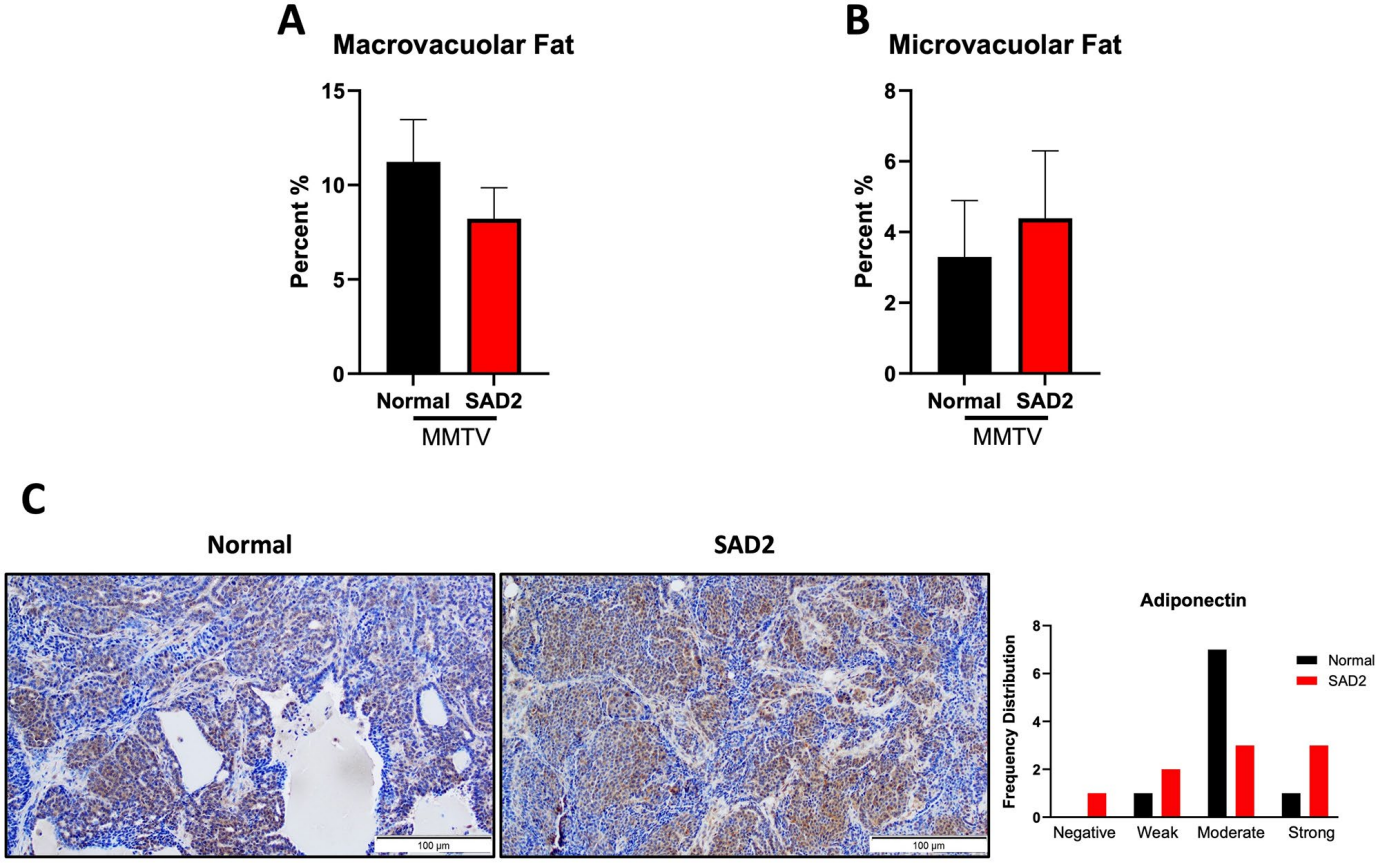
Tumor characterization in MMTV mice. MMTV tumor tissues sampled across different mice (*n* = 17 normal chow and *n* = 18 SAD2) were scored for microvacuolar and microvacuolar fat and evaluated for adiponectin levels. (**A**) Percentage of macrovacuolar fat in MMTV tumors. (**B**) Percentage of microvacuolar fat in the tumor tissues of MMTV tumors. (**C**) A subset of tumors (*n* = 9 normal chow and *n* = 9 SAD2) sampled across different mice were subjected to immunohistochemistry and adiponectin staining. Staining was scored by pathologists, and GraphPad Prism was used to graph the values, which are displayed as the means ± SEMs. The images are representative, and the scale bar is 100 μm

Tumors from MMTV mice were evaluated by a pathologist for the percentage of macrovacuolar and microvacuolar fat, necrosis, vascular proliferation, and fibrosis (Fig. 7 and Suppl. Fig. 6). A decrease in macrovacuolar fat was found in the SAD2 group, which correlated with an increase in microvacuolar fat; however, neither trend was statistically significant (Fig. 7A and B). The levels of adiponectin, a protein hormone released by adipose tissue, were measured using immunohistochemistry and scored based on intensity. The SAD2 group was found to have increased negative, weak and strong adiponectin levels. However, these parameters decreased in the moderate group after the consumption of the SAD2 diet [33] (Fig. 7C and Suppl. Fig. 7). Gene expression levels showed a similar but nonsignificant trend for adiponectin (Suppl. Fig. 7). Additionally, we observed an increase in the percentage of necrosis and fibrosis within the tumors of the SAD2-fed mice (Suppl. Fig. 6).

**Fig. 8:**
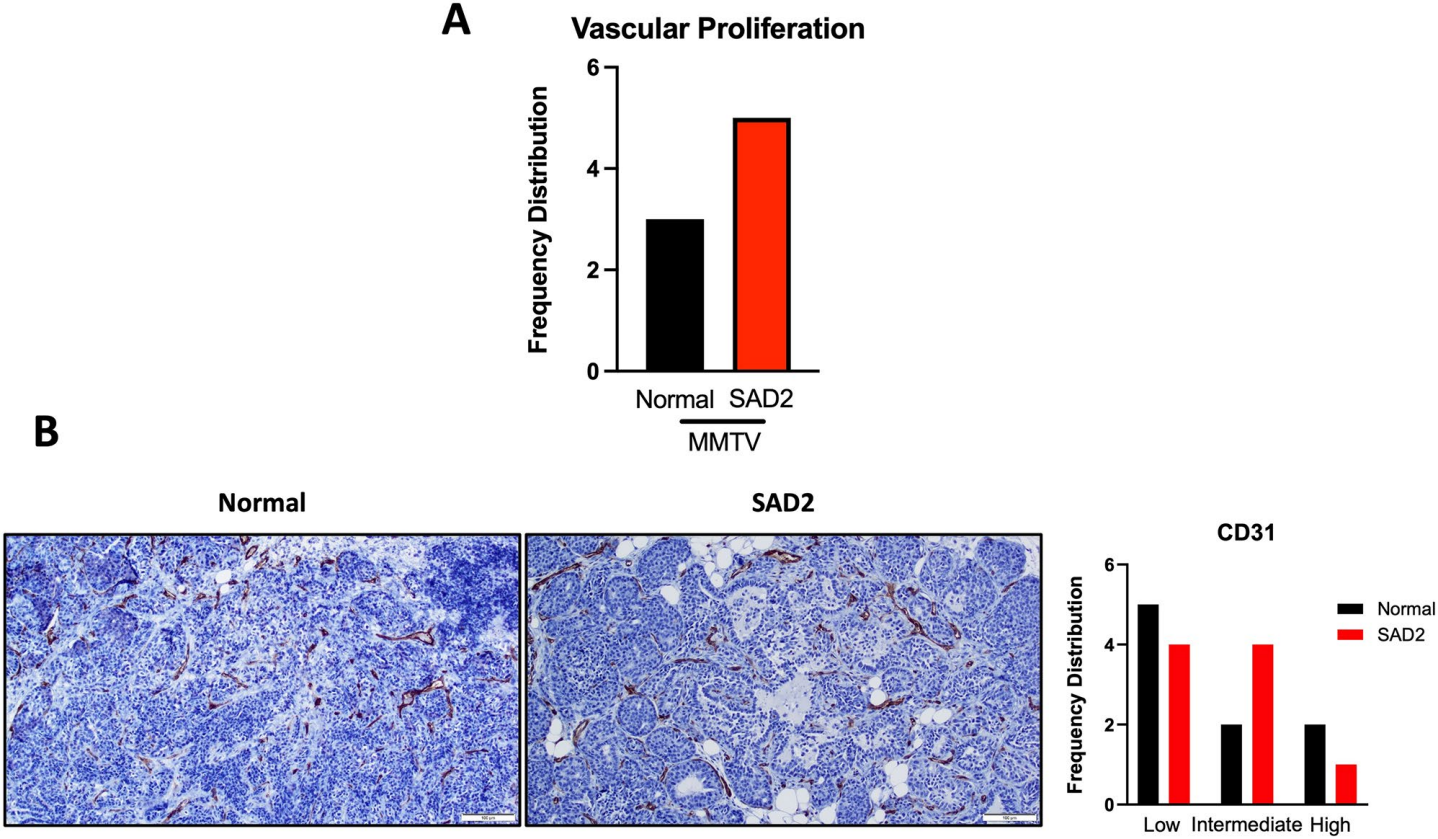
Changes in vascular proliferation in MMTV tumors. (**A**) MMTV tumor tissues sampled across different mice (*n* = 17 normal chow and *n* = 18 SAD2) were scored for vascularization. The frequency distribution graph shows the number of tumors with vascular proliferation. (**B**) Immunohistochemistry was performed to evaluate cluster of differentiation 31 (CD31) levels in MMTV tumors from separate mice (*n* = 9 normal chow and *n* = 9 SAD2), which were scored based on the presence of staining by pathologists. The values are displayed as the means ± SEMs. All the graphs were plotted via GraphPad Prism. The images are representative, and the scale bar is 100 μm

**Fig. 9:**
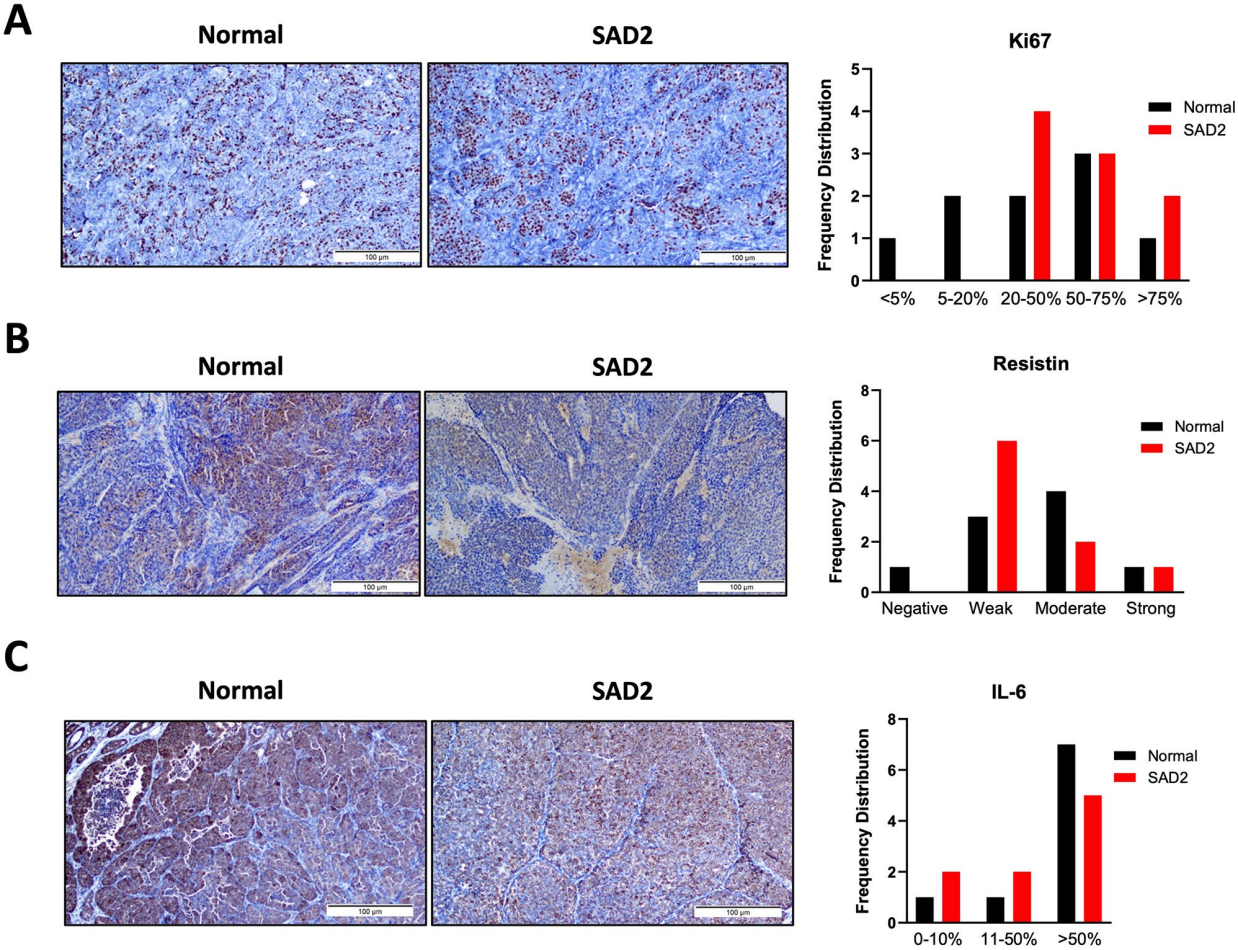
Tumor proliferation changes in MMTV tumors. Immunohistochemistry was performed on a subset of MMTV tumor tissues from individual mice to evaluate the levels of the Kiel 67 (Ki67), resistin and interleukin 6 (IL-6) antigens (*n* = 9 normal chow and *n* = 9 SAD2). (**A**) Ki67 levels were graded by pathologists based on the percentage of stained cells in MMTV tumors. (**B**) Resistin levels were scored based on staining intensity in each tumor sample. (**C**) IL-6 levels were scored based on the staining intensity of each tumor sample. The values were graphed via GraphPad Prism and are presented as the means ± SEMs. The images are representative, and the scale bar is 100 μm

Vascular proliferation was also evaluated (Fig. 8). An increase in the number of tumors that exhibited vascular proliferation was detected in SAD2 mice (Fig. 8A). The cluster of differentiation 31 (CD31), a vascular marker, was found to decrease at low and high intensities but increase at moderate levels (Fig. 8B). Additionally, the antigen Kiel 67 (Ki67), a cellular marker for proliferation, was investigated next using immunohistochemistry (Fig. 9). Tumors were scored based on the percentage of cells expressing Ki67, and higher Ki67 scores were observed in tumors from SAD2-fed mice than in those from control mice (Fig. 9A). However, the levels of β-catenin, a signaling protein involved in cell proliferation, increased at 51–75% but decreased at > 75% in the SAD2 diet (Suppl. Fig. 8). The levels of resistin and interleukin 6 (IL-6) were measured using immunohistochemistry (Fig. 9, Suppl. Fig. 8). Resistin is an inflammatory cytokine that plays a role in tumor proliferation and energy metabolism [34]. Compared with control mice, SAD2-fed mice presented slightly lower levels of resistin in tumors but higher levels in the plasma (Figs. 2B and 9B). IL-6, a marker of cell invasiveness and metastasis, was also slightly increased in tumors in the 0–0–50% group, whereas fewer tumors in the SAD2 group exhibited > 50% staining [35] (Fig. 9C). No significant changes in the gene expression levels of resistin and IL-6 were detected (Suppl. Fig. 9). These findings suggest that the SAD2 diet may alter tumor characteristics in MMTV mice, with changes related to tumor proliferation.

### SAD2 increases glycation products and DNA damage in MMTV tumors

**Fig. 10:**
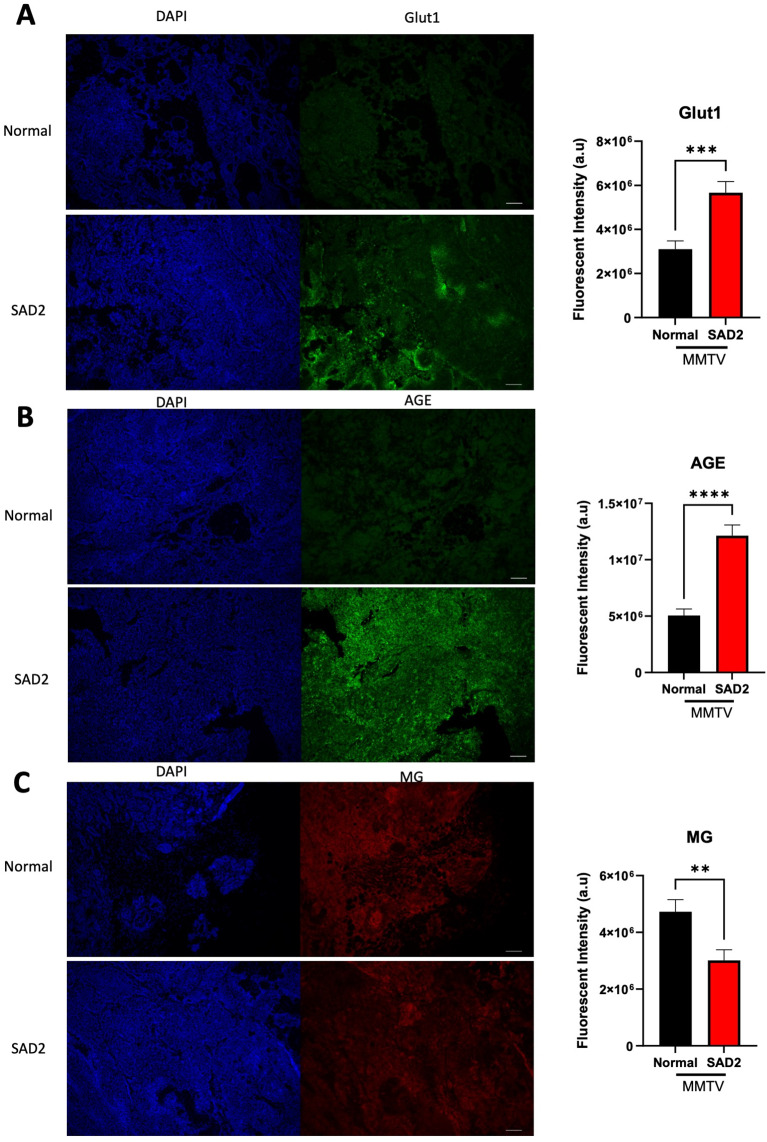
Increased levels of GLUT1 and its glycation products in SAD2-fed MMTV mice. Immunofluorescence was performed in a subset of MMTV tumor tissues from individual mice to evaluate (**A**) glucose transporter 1 (GLUT1), (**B**) advanced glycation end products (AGEs), and (**C**) methylglyoxal (MG) levels (*n* = 8 normal chow and *n* = 10 SAD2). A binary threshold was used to measure the fluorescence intensity, and GraphPad Prism was used to plot values, which are displayed as the means ± SEMs. Significance is displayed as follows: **p < 0.01; ***p < 0.001; ****p < 0.0001. The images displayed are representative, and the scale bar is 100 μm

To characterize the tumors further, we assessed changes in glucose transporter (Glut1) expression and the levels of advanced glycation end products (AGEs) and methylglyoxal (MG) in MMTV tumors using immunofluorescence (Fig. 10). With the increase in carbohydrates and fat within the SAD2 diet and the observed increase in blood glucose, the levels of Glut1 may increase along with glycation products such as AGEs and MG [36]. These products are created through a nonenzymatic reaction of excess sugars with proteins or lipids. They have been implicated in the development or worsening of degenerative diseases, such as diabetes [36]. Glut1 expression increased significantly in the SAD2 group (Fig. 10A). AGE levels also increased significantly in the SAD2 tumors (Fig. 10B). However, a significant decrease in MG was observed (Fig. 10C).

**Fig. 11:**
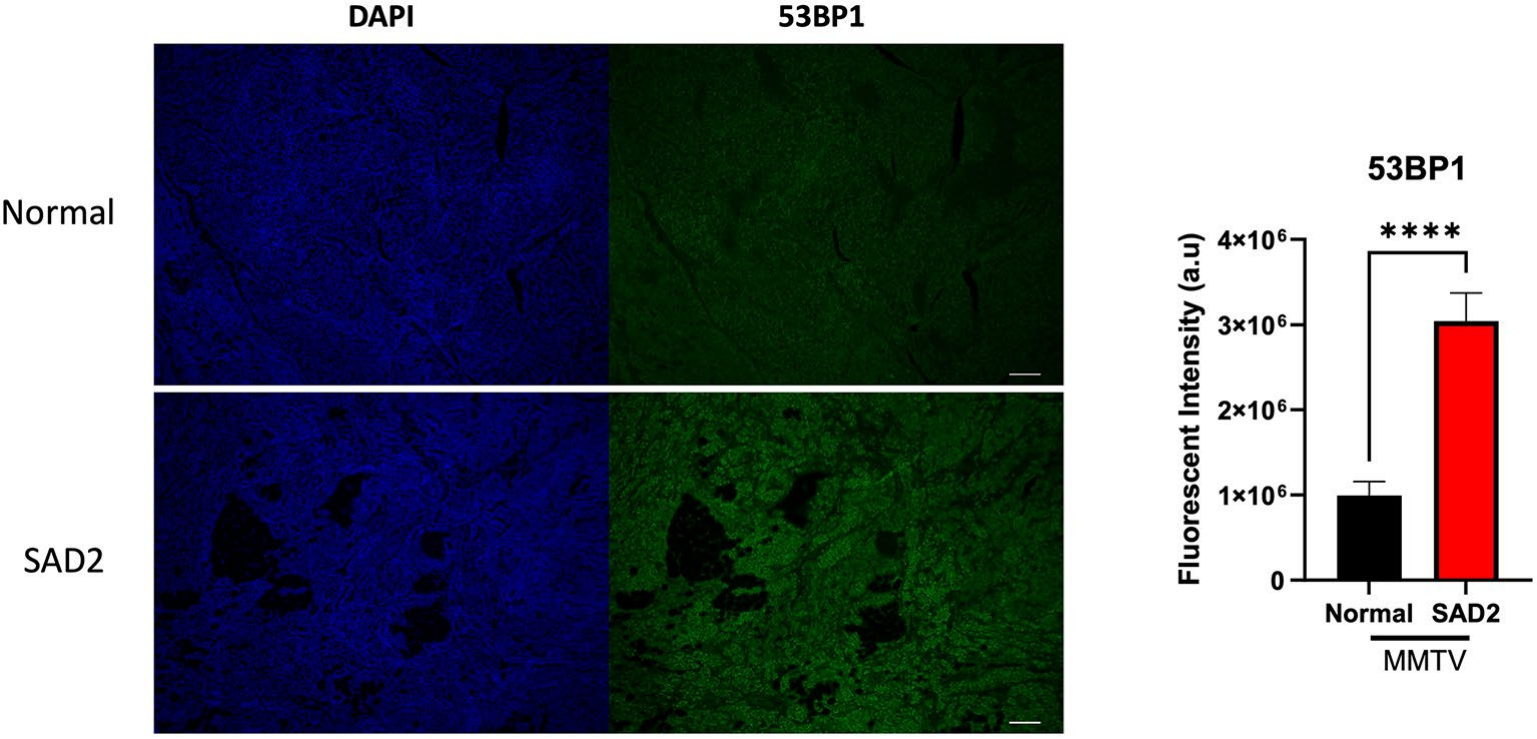
SAD2 diet promotes double-strand breaks in MMTV tumors. Immunofluorescence staining was used to evaluate the levels of the tumor suppressor p53 binding protein 1 (53BP1) in a subset of MMTV tumors from individual mice (*n* = 8 normal chow and *n* = 10 SAD2). A binary threshold was used to measure the fluorescence intensity for each tissue. The values are displayed as the means ± SEMs in GraphPad Prism. Significance is displayed as follows: ****p < 0.0001. The images displayed are representative, and the scale bar is 100 μm

**Fig. 12:**
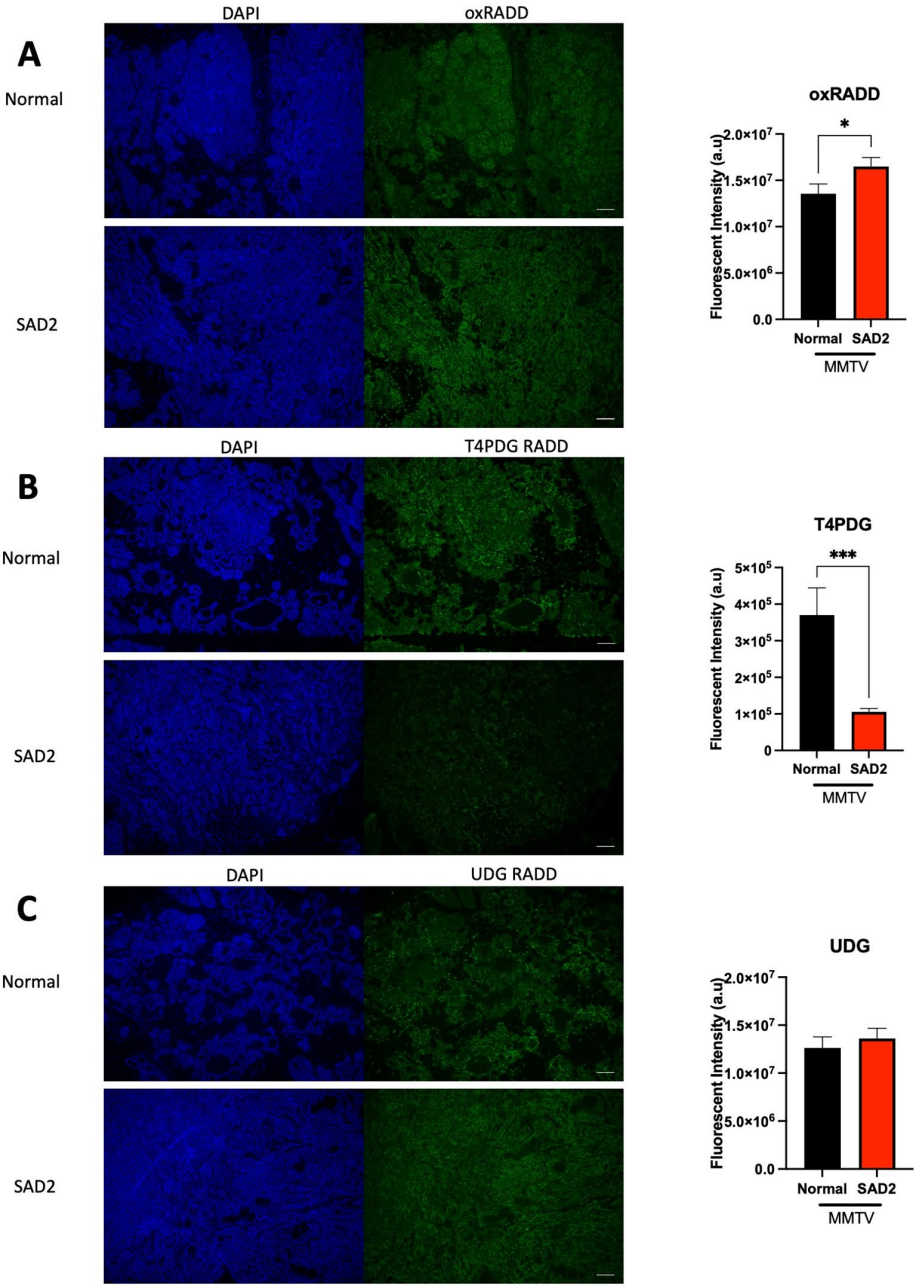
DNA lesion profile is altered in SAD2-fed MMTV tumors. The Repair Assisted Damage Detection (RADD) assay measures various types of lesions in MMTV tumors from individual mice via different cocktails (*n* = 7 normal chow and *n* = 8 SAD2). (**A**) Oxidative lesions (oxRADD), (**B**) crosslink-type lesions (T4PDG), and (**C**) uracil lesions (UDG). Adduct levels were measured using immunofluorescence, and a binary threshold was used to determine the fluorescence intensity of the tumor tissue. The data are displayed as the mean ± SEM of the fluorescence intensity and were plotted via GraphPad Prism. Significance levels are as follows: *p < 0.05; ***p < 0.001. The images are representative, and the scale bar is 100 μm

Next, we examined the DNA damage marker tumor suppressor p53 binding protein 1 (53BP1) in these tumors. 53BP1 is a double-strand break indicator, and a significant increase in 53BP1 levels was observed in SAD2 tumors (Fig. 11). Owing to the increased DNA damage observed in MMTV tumors, we used the DNA damage detection assay Repair Assist Damage Detection (RADD) to examine the levels of different types of DNA lesions within the tumors. Oxidative lesions (oxRADD), uracil lesions (UDG) and crosslink-type lesions (T4PDG) were measured (Fig. 12, Suppl. Fig. 10 for Ig control). Significantly higher levels of oxidative lesions were observed in SAD2 tumors, which was consistent with the increase in AGE levels (Fig. 12A). Conversely, the number of crosslink-type lesions was significantly lower in the SAD2 group than in the control group (Fig. 12B), indicating that the SAD2 diet may enhance the repair of these lesion types. The levels of uracil lesions were similar between the two groups (Fig. 12C), suggesting the absence of mitochondrial dysfunction. We also examined the mitochondrial content via mitochondrial markers and found that the levels of mitochondria were similar among the groups (Suppl. Fig. 10).

### SAD2 affects the levels of DNA repair proteins in MMTV mice

**Fig. 13:**
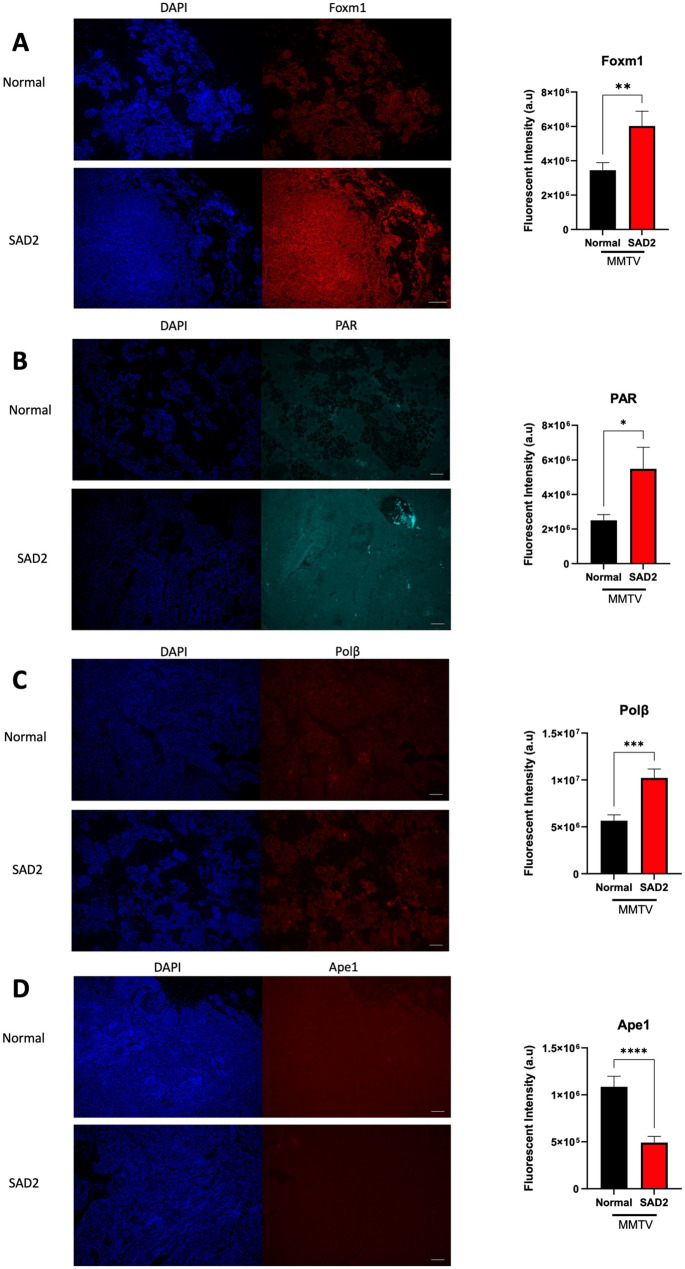
SAD2 diet promotes changes in DNA damage and response markers. Immunofluorescence was used to measure various types of DNA damage and response markers in MMTV tumors from individual mice (*n* = 7 normal chow and *n* = 8 SAD2). (**A**) Forkhead box M1 (Foxm1), (**B**) poly-ADP ribose (PAR), (**C**) DNA polymerase beta (Polβ), and (**D**) apurinic/apyrimidinic endonuclease 1 (Ape1). The intensity levels were evaluated using a binary threshold to detect the fluorescence intensity. The graphs are displayed as the means ± SEMs via GraphPad Prism, and the significance levels are as follows: *p < 0.05; **p < 0.01; ***p < 0.001; ****p < 0.0001. The images are representative, and the scale bar is 100 μm

Next, we analyzed DNA repair proteins in MMTV tumors using immunofluorescence. Forkhead box M1 (Foxm1) is a transcription factor implicated in DNA repair in response to DNA damage and oxidative stress and can regulate glycolysis and energy production in various types of tumors [37]. Compared with normal chow, SAD2 tumors were significantly more common (Fig. 13A). The level of poly(ADP-ribose) (PAR), which is generated to signal the repair of base lesions and strand breaks by poly(ADP-ribose) polymerase 1 (Parp1), was also significantly increased in SAD2 tumors (Fig. 13B). DNA polymerase beta (PolB) levels were also assessed. A significant increase in the number of tumors was detected in SAD2-fed mice (Fig. 13C). Interestingly, apurinic/apyrimidinic endonuclease 1 (Ape1) was significantly decreased in the SAD2-fed mice (Fig. 13D). The gene expression of Foxm1, PolB, and Apex1 revealed similar trends. However, they were not significant (Suppl. Fig. 11). These findings are consistent with the increase in oxidative lesions in SAD2 tumors and suggest altered base excision repair due to low Ape1 levels and increased lesion content within these tumors.

## Discussion

The prevalence of both breast cancer and obesity has increased among women. In patients with breast cancer, obesity increases the risk of recurrence or death by 30% [38]. Recent studies have used high-fat diets (HFDs) or Western-style diets to model these conditions and understand tumorigenic effects in murine models. These studies also identified key markers, including increased body mass, adiposity, and inflammatory cytokines. However, the mechanistic interactions between breast cancer and obesity are not fully understood, and realistic dietary models are lacking.

Here, we examined a modified standard American diet (SAD2) as a model to induce obesity and alter breast cancer pathogenesis. The diet composition was repeated from Wiggins et al. 2022, where the SAD2 diet was originally described, and exposure to the diet produced type II diabetes-like phenotypes in the CD-1 mice [20]. The diet contained 35% fat, comparable to Western-style diets, and contained milk fat and vegetable shortening [20–22]. We investigated its effects on spontaneous breast cancer MMTV-PyMT mice and their FVB strain controls [39].

FVB and MMTV mice were fed normal chow or SAD2 for 12 weeks. Increased body mass and adiposity were characterized in both mouse models (Figs. 1 and 2). While there is some evidence of resistance to diet-induced obesity within this strain, the increases in body weight and adipose deposits were consistent with reports from HFDs studies (Fig. 2) [15, 16, 41]. The SAD2 diet al.so increased blood glucose levels in the fasted state for the FVB mice at 12 weeks (Suppl. Fig. 3) and in the MMTV between 10 and 12 weeks (Fig. 3). Mice were only fasted for 4 h, which may have impacted the significance of blood glucose changes. Fasting times between 4 and 6 h have been reported to have similar impacts on insulin tolerance [26]. Although the levels did not reach the hyperglycemic index for diabetes within the short diet period used, SAD2 did increase blood glucose without glucose or sucrose supplementation. SAD2 consumption in CD-1 mice for 30 weeks induced hyperglycemia [20], suggesting that a longer diet interval could induce hyperglycemia.

SAD2 also significantly increased circulating resistin levels, which is associated with more aggressive and metastatic tumors in patients with breast cancer (Fig. 4) [42]. We also observed elevated levels of circulating GIP and leptin (Fig. 4). Increased leptin is associated with increased body fat mass and promotes breast cancer cell growth by hindering proapoptotic signaling pathways [43, 44]. However, the role of GIP in breast cancer remains unclear. It regulates circulating glucose and insulin secretion and has been identified as a therapeutic target for neuroendocrine tumors [45]. The new focus on GLP-1 agonists raises the possibility that GLP-1/GIP agonists may reduce tumor progression and associated lymphedema [46–48], although these effects are still under investigation. Given the short diet interval and resistance of the FVB strain, we observed several metabolic and physiological effects of the SAD2 diet, suggesting its utility in breast cancer studies.

Compared with normal chow-fed mice, SAD2-fed mice presented faster tumor growth and greater tumor multiplicity (Fig. 6B). We also observed reduced survival in these mice, with a combined tumor volume at or exceeding 1500 mm^3^ or humane endpoints used as the study endpoint (Fig. 6A). Some HFD studies have used a longer diet period (up to 16 weeks) and a much greater tumor volume (2000–3000 mm^3^) [15], which could be implemented for further SAD2. We selected a more conservative endpoint to reduce animal loss to mobility issues from high and low mammary region tumors (under limbs) and skin necrosis. We still observed significant tumor growth under the limbs. Our study design was similar to that of Sundaram et al. and Velazquez et al., who employed 40–45% higher fat diets for 8 weeks [16, 41]. Despite the conservative tumor volume endpoint, we observed significant differences between the SAD2 and normal chow diet-fed tumors.

We also observed increased Glut1 expression and AGEs within SAD2 tumors (Fig. 10). Increased glycolytic demands in cancer cells, referred to as the “Warburg effect,” increase the need for glucose uptake through Glut1. Glut1 expression is elevated in breast cancer, with the TCGA dataset showing increased levels of *GLUT1* (*SLC2A1*) in luminal, HER2-positive, and triple-negative breast cancer [49]. Although no association between GLUT1 expression and diabetic or obese breast cancer patients has been reported, elevated GLUT1 expression is associated with more aggressive cancers and poorer prognosis [50]. An HFD decreased Glut1 expression in the brain and normal mammary during lactation [51, 52]. However, there are no reports where an HFD increases Glut1 expression, so a unique aspect of the SAD2 diet is increased Glut1 expression, which recapitulates the trends observed in 60% of breast cancer patients [53].

In line with the increased Glut1 levels, we also observed increased Foxm1, an oncogenic transcription factor (Fig. 13). FOXM1 is linked to the expression of GLUT1 in hepatocellular carcinoma and breast cancer [54]. FOXM1 is overexpressed in a number of cancers, including breast cancer, and is associated with aggressive disease and poor prognosis [55]. FOXM1, GLUT1, and STAT3, other oncogenic transcription factors, are commonly associated with hepatocellular carcinoma, where activated STAT3 is proposed to regulate the expression of FOXM1 and GLUT1 [56–58]. However, a study using a PROTAC degrader of FOXM1 revealed decreased GLUT1 expression in hepatocellular carcinoma and breast cancer models [54]. The use of SAD2 resulted in increased expression along this axis, which may provide new opportunities for in vivo drugs that target FOXM1, GLUT1, or STAT3 [55, 59, 60].

Additionally, we observed increased AGEs produced by diabetic pathologies (Fig. 10) [61]. These metabolites are produced by Maillard reactions with DNA, lipids, and amino groups. AGEs in the serum are associated with metastatic disease and increased invasion and migration of breast cancer cells [62]. An HFD can result in elevated levels of AGEs in the liver and muscle; however, this effect has not been observed in breast tumors. Therefore, another unique feature of the SAD2 diet is elevated AGE levels. The reduced methylglyoxal levels observed within tumors could reflect their rapid conversion to AGEs or suggest that excess methylglyoxal is excreted. We did not measure plasma methylglyoxal levels, but this may be of interest for future studies. Increased glycation stress is coupled with elevated oxidative lesions in the tumors of SAD2-fed mice, as measured by RADD (Fig. 12).

Given the increased oxidative lesion levels in the SAD2-fed mice, we examined selected proteins in the base excision repair (BER) pathway and observed increased Polβ and PAR, which are produced by Parp1 for signal repair at base lesions and strand breaks (Fig. 13) [63, 64]. Elevated levels of PAR are associated with dysfunctions in the BER and homologous recombination pathways [63, 65, 66]. While Polβ is elevated, Ape1, which acts upstream of Polβ, is decreased in SAD2 mice (Fig. 13). BER is initiated when damaged bases are recognized and excised by various DNA glycosylases [67]. DNA glycosylases bind and cleave the N-glycosidic bond of the damaged base, leaving an abasic site that lacks the sugar moiety. The abasic site is recognized by APE1, which incises the DNA backbone to create a single nucleotide gap and 5’-deoxyribose flap for Polβ removal [67]. While some bifunctional DNA glycosylases can also create a single nucleotide gap with different end chemistries, Ape1 typically plays a critical role in abasic site removal for BER repair [68]. Low levels of Ape1 in SAD2 mice may slow BER, increasing the retention of mutagenic oxidative lesions within the DNA (Fig. 12).

Interestingly, we also observed a decrease in T4 PDG-recognized lesions between normal chow-fed and SAD2 diet-fed tumors (Fig. 12). We previously associated decreased lesion levels with increased DNA repair capacity for these lesions [29, 69, 70]. T4 PDG canonically recognizes cyclobutane pyrimidine dimers (CPDs) and 6 − 4 photoproducts repaired by nucleotide excision repair (NER). The decrease in these lesions may suggest increased DNA repair by the NER pathway, which could also indicate increased resistance to platinum-based therapies. Additionally, methylglyoxal-DNA lesions are proposed to be removed by BER and NER, so elevated function in these pathways could also reduce the detected levels of methylglyoxal (Fig. 10) [71]. While functional testing of the BER and NER pathways would be necessary in future studies, FOXM1 regulates the expression of several DNA repair proteins, including POLβ, DNA polymerase ε, EXO1, and NBS1 [55]. Therefore, dysregulation of this axis by the SAD2 diet could alter the DNA repair capacity and influence therapeutic efficacy.

While we observed significant impacts from the SAD2 diet on adiposity and DNA damage and repair, other dietary-linked effects, e.g., changes in cytokines or chemokines, were more modest, limiting the ability to interpret systemic impacts of the diet compared to HFD. These modest effects may be attributable to the FVB/N background, which is often considered obesity-resistant [40]. A limitation of this work is that these mice may not be as susceptible to the SAD2 diet effects and show a more modest inflammatory phenotype [20–22]. Future studies should expand the SAD2 characterization to MMTV-PyMT mice with a C57BL/6 background, which may better reflect obesity-associated breast cancers in patients with inflammatory signatures.

There are several other limitations to this study. We selected the normal chow and SAD2 diets from Wiggins et al. to observe how the type II diabetes-like phenotypes observed in that study impacted tumor growth [20]. The diet pairs are not isonitrogenous and have distinct ingredients (Table 1). Future studies could use the AIN93 standard diet to offer a more comparable protein level of 15.4–13.7%, but the SAD2 diet will have distinct fat and buffering ingredients to maintain its higher fat content. Another limitation of our study compared to the work of Wiggins et al. is the short study duration for the MMTV-PyMT mice employed. Pre- and post-menopausal status have differing effects on breast cancer development and progression, which cannot be accounted for with this rapid model and the short diet duration employed. Further, we limited the diet period to 12 weeks to prevent excessive tumor burden instead of using a fixed diet period of 16 weeks used for some HFD work. Employing this short study duration may have impacted the significance of some of the results, but the hallmarks we see regarding adiposity and DNA damage are still significant. Shifting away from the MMTV-PyMT mice to syngeneic or xenograft models would allow longer diet durations of 12–30 weeks that may enhance the inflammatory impact of the SAD2 diet and allow age-dependent hormonal changes to be investigated [15, 20, 72].

Despite these limitations, we observed significant changes in the expression of the oncogenic transcription factor Foxm1 and glucose transporter Glut1, which are observed in a significant number of breast cancers and have not been previously linked to dietary changes [73]. We also determined that even short-term exposure to the SAD2 diet induced DNA and protein damage with elevated oxidative and glycation damage, which are expected for diabetic and obese patients [74, 75]. Our results suggest that the SAD2 diet may recapitulate the dietary effects observed in patient samples within mouse models and reflect early obesity and diabetes phenotypes.

## Conclusions

Using an American-style diet (SAD2), we demonstrated that MMTV-PyMT mice fed this diet increased weight and adiposity and formed tumors with increased oxidative DNA damage and AGEs compared to normal chow. In addition, the SAD2-fed mice tumors showed increased expression of oncogenic transcription factor Foxm1 within a relatively short diet interval used in this work. Unlike previous studies with HFD and western diet, the changes in inflammatory cytokines and other metabolic factors were mild using the short diet period, yet the SAD2 diet already impacted the tumor initiation and growth. These data suggest that the SAD2 diet may offer better insight into mechanisms that promote breast cancer aggressiveness and resistance to therapy in overweight and obese American women.

## Electronic supplementary material

Below is the link to the electronic supplementary material.

Supplementary Material 1

## Data Availability

All the data generated or analyzed during this study are included in this published article and its supplementary information files.

## Abbreviations

DAB: 3,3’-Diaminobenzidine
DAPI: 4′,6-Diamidino-2-Phenylindole
AGEs: Advanced Glycation End Products
ASE: Antibody Signal Enhancer
β-catenin: Beta-catenin
Ape1: Apurinic/Apyrimidinic Endonuclease 1
RANTES: chemokine ligand 5
MIG: chemokine ligand 9
CD31: Cluster of Differentiation 31
C-peptide 2: Connecting peptide 2
Polβ: DNA polymerase beta
Endo IV: Endonuclease IV
Endo VII: Endonuclease VIII
EDTA: Ethylenediaminetetraacetic acid
FPG: Fapy-DNA Glycosylase
Foxm1: Forkhead box M1
GLP-1: Glucagon-Like Peptide 1
GIP: Glucose-Dependent Insulinotropic Polypeptide
Glut1: Glucose Transporter 1
G-CSF: Granulocyte Colony-Stimulating Factor
HFD: High-Fat Diet
HER: Human Epidermal Growth Factor 2
IACUC: Institutional Animal Care and Use Committee
IFNγ: Interferon gamma
IL-1α: Interleukin 1 alpha
IL-1β: Interleukin 1 beta
IL-2: Interleukin 2
IL-3: Interleukin 3
IL-4: Interleukin 4
IL-5: Interleukin 5
IL-6: Interleukin 6
IL-7: Interleukin 7
IL-9: Interleukin 9
IL-10: Interleukin 10
IL-12p40: Interleukin 12 β
IL-12p70: Interleukin 12
IL-13: Interleukin 13
IL-15: Interleukin 15
IL-17 A: Interleukin 17 α
IP-10: Interferon Gamma-Induced Protein 10 ()
HE: Hematoxylin and Eosin
HRP: Horse Radish Peroxidase
KC: keratinocyte chemoattractant
Ki67: Kiel 67
LIF: Leukemia Inhibitory Factor
LIX: Lipopolysaccharide-Induced Chemokine
M-CSF: Macrophage Colony-Stimulating Factor
MIP-1α: Macrophage Inflammatory Protein 1 alpha
MIP-1β: Macrophage Inflammatory Protein 1 beta
MIP-2: Macrophage Inflammatory Protein 2
MG: Methylglyoxal
SAD2: Modified Standard American Diet
MCP-1: Monocyte Chemotactic Protein-1
MMTV-PyMT: Mouse Mammary Tumor Virus-Polyoma Middle Tumor-Antigen
NBF: Neutral Buffered Formalin
ON: Overnight
oxRADD: oxidative lesions RADD
PP: Pancreatic Polypeptide
PYY: Peptide Tyrosine Tyrosine
PBS: Phosphate-Buffered Saline
PAR: Poly(ADP-Ribose)
RADD: Repair Assisted Damage Detection
RT: Room Temperature
SAD: Standard American Diet
T4PDG: T4 Pyrimidine Dimer Glycosylase
TBS: Tris-Buffered Saline
TBST: TBS with Tween-20
TNFα: Tumor Necrosis Factor-α
53BP1: Tumor suppressor p53 binding protein 1
UAB: University of Alabama at Birmingham
UDG: Uracil DNA Glycosylase
VEGF: Vascular Endothelial Growth Factor
