# Supplementary material for: Short-term consumption of the modified standard American diet perturbed the metabolic balance and altered DNA damage in MMTV-PyMT transgenic mice
Source: Breast Cancer Res. 2025 Jul 25;27:138. doi: 10.1186/s13058-025-02075-w (PMC12296626; doi:10.1186/s13058-025-02075-w)
Supplement: Supplementary file 1 — Supplementary Material 1 [file 13058_2025_2075_MOESM1_ESM.pdf]

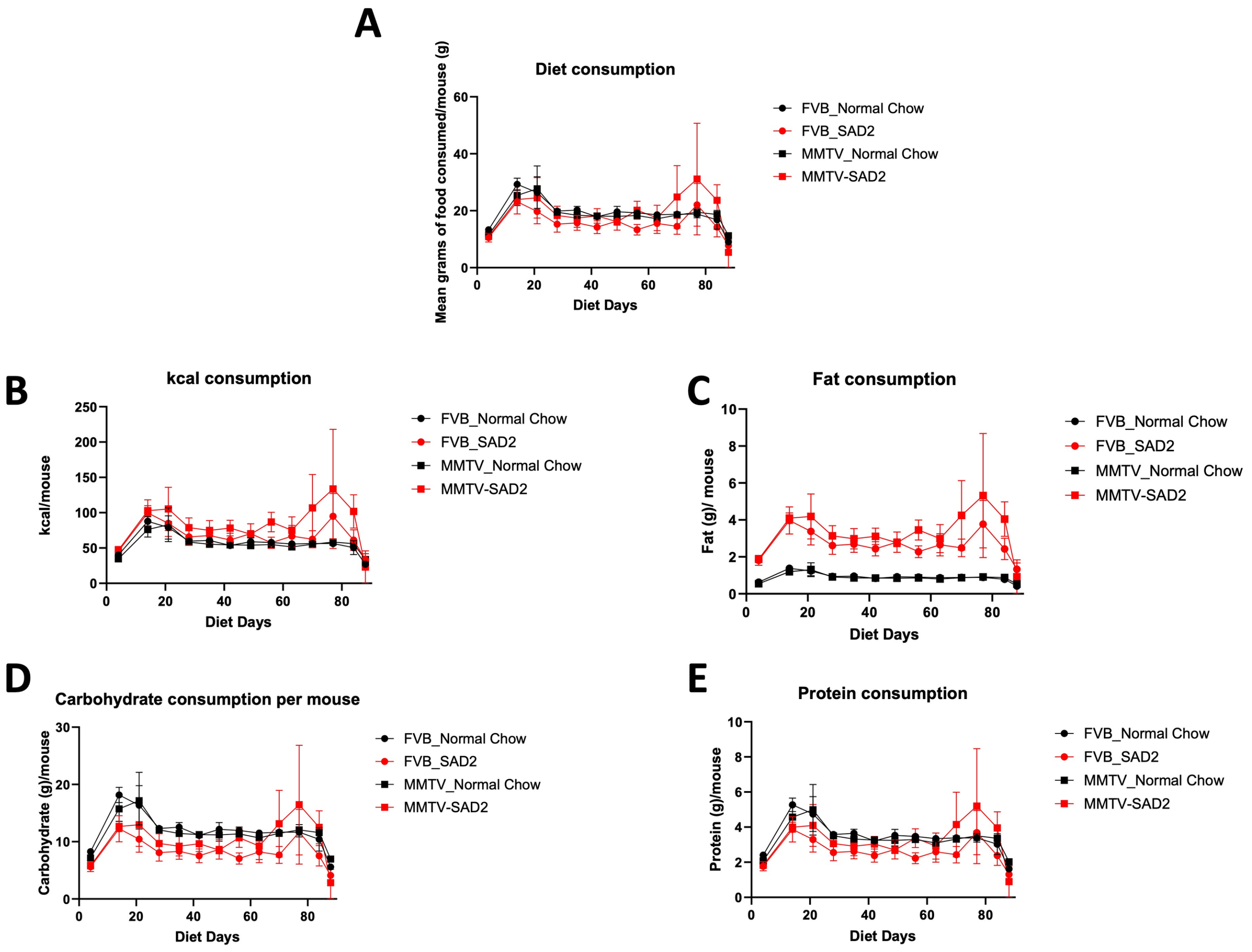


**Suppl. Fig. 1. Overall diet consumption in FVB and MMTV mice.** Food consumption was monitored and weighed weekly throughout the study in FVB (n=11 normal chow and n=12 SAD2 at start) and MMTV mice (n=12 normal chow and n=12 SAD2 at start). (A) Average grams (g) consumed per mouse. (B) Kilocalories (kcal) per mouse were calculated. (C) Fat consumption per mouse in g. (D) Carbohydrate consumption per mouse in g. (E) Protein consumption per mouse in g. GraphPad Prism was used to graph the values presented as the means ± SEMs. At day 84, mice numbers dropped: FVB mice (n=9 normal chow and n=12 SAD2) and MMTV mice (n=5 normal chow and n=3 SAD2).

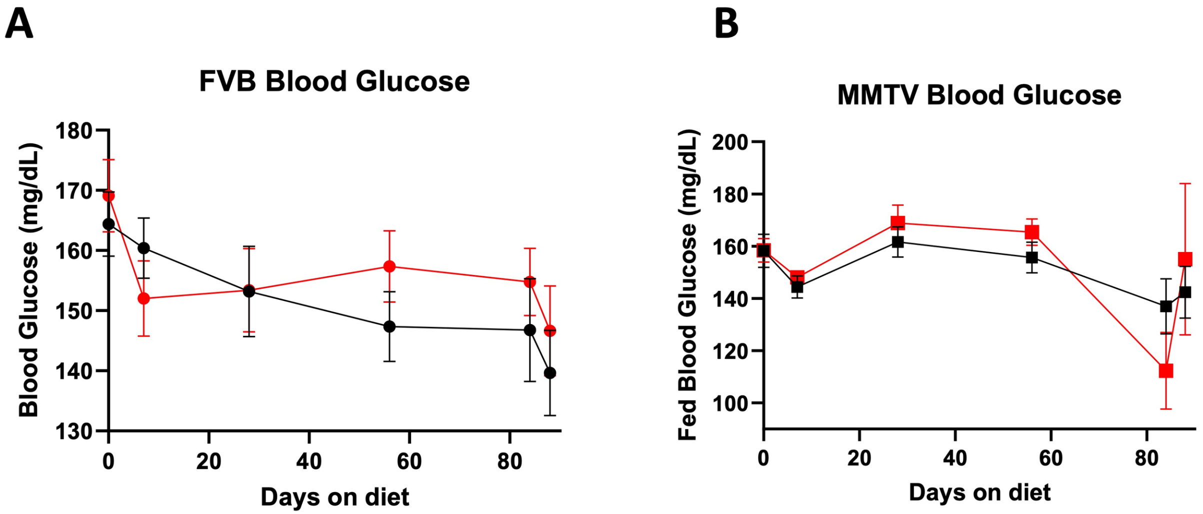


**Suppl. Fig. 2. Glucose levels in FVB and MMTV mice throughout the study.** Blood glucose levels at weeks 1, 4, 8, and 12 were measured via the Contour Next Blood Glucose Monitoring System. Blood glucose levels in (A) FVB mice (n=10 normal chow and n=12 SAD2 at start) and (B) MMTV mice (n=10 normal chow and n=12 SAD2 at start). The values are displayed as the means ± SEMs and were analyzed via GraphPad Prism. At day 84, mice numbers dropped: FVB mice (n=9 normal chow and n=12 SAD2) and MMTV mice (n=5 normal chow and n=3 SAD2).

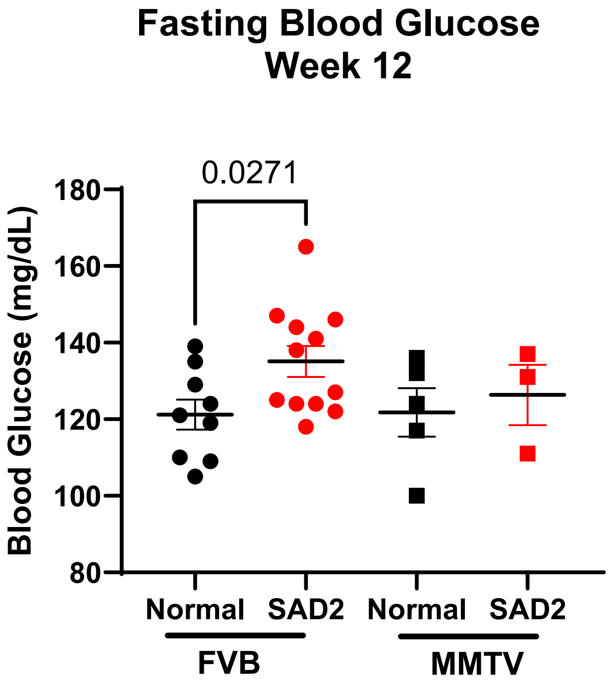


**Suppl. Fig. 3. Fasting blood glucose levels at week 12 in FVB and MMTV mice.** Fasted glucose measurement was performed at week 12 in FVB and MMTV mice via a Contour Next Blood Glucose Monitoring System. (A) Blood glucose levels in FVB mice (n=9 normal chow and n=12 SAD2). (B) Blood glucose levels in MMTV-treated mice (n=5 normal chow and n=3 SAD2). The data are displayed as the means ± SEMs via GraphPad Prism.

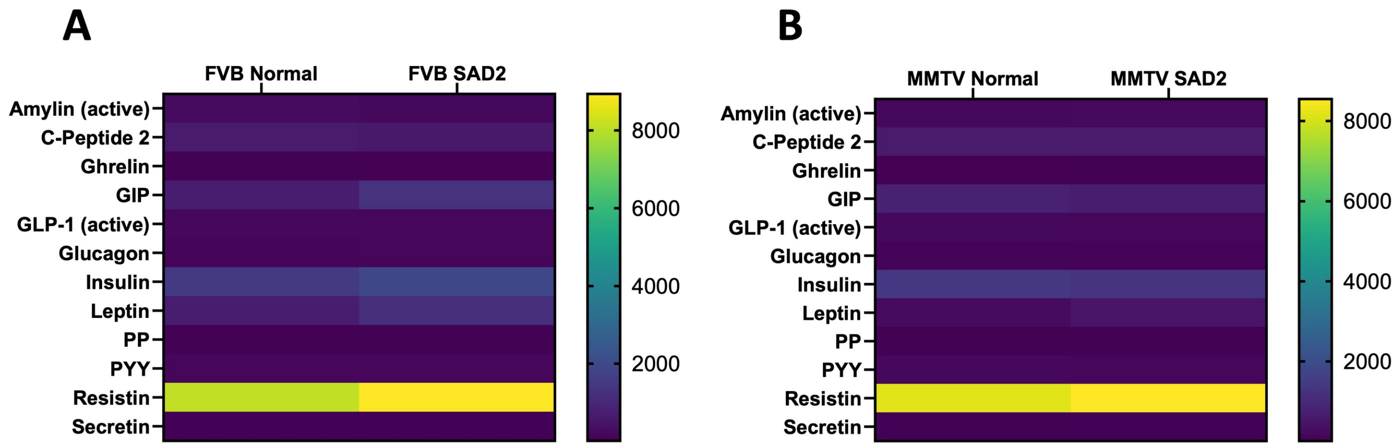


**Suppl. Fig. 4. Metabolic cytokines and hormones in FVB and MMTV mice.** Plasma was obtained from a subset of FVB (n=6 normal and n=5 SAD2) and MMTV (n=10 normal and n=10 SAD2) mice before termination to evaluate metabolic cytokines and hormones using a metabolic discovery array panel in EVE technologies. A heatmap was used to plot the various metabolic cytokines and hormones in (A) FVB and (B) MMTV mice. GraphPad Prism was used to graph the values as medians.

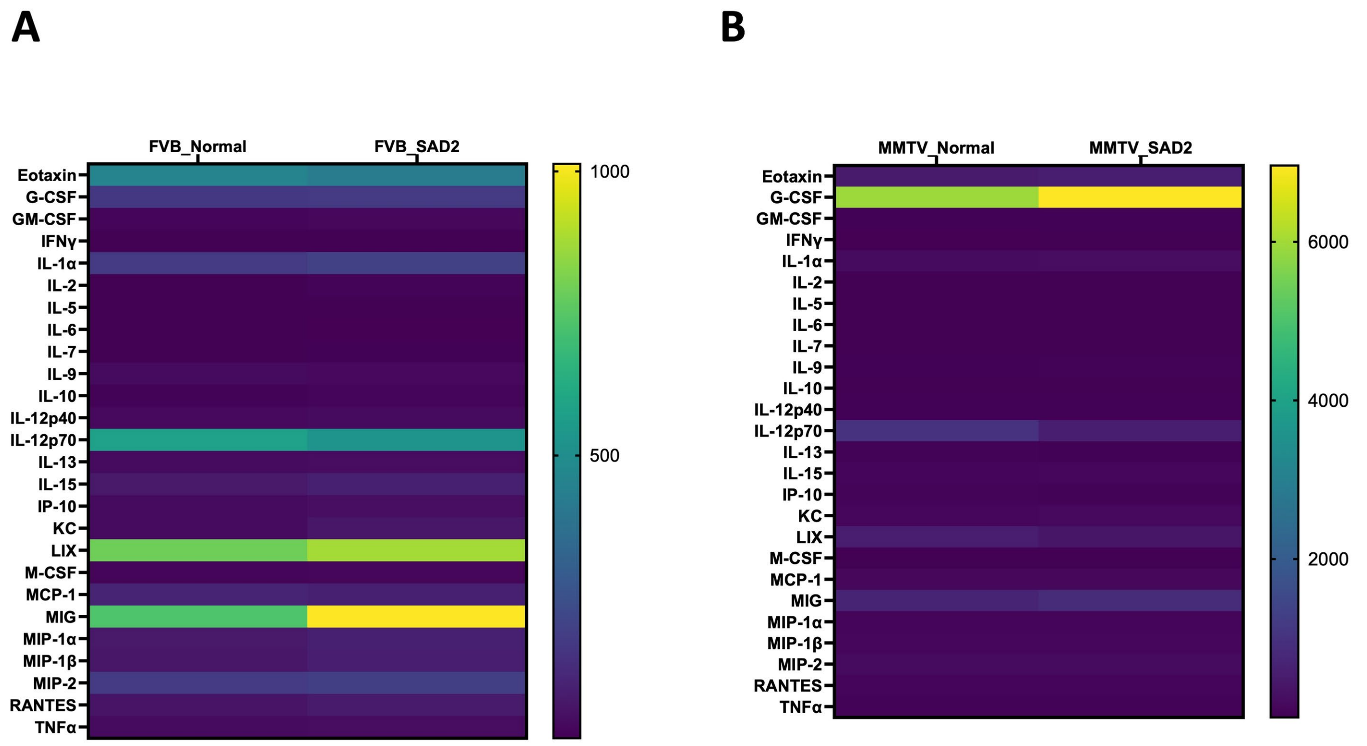


**Suppl. Fig. 5. Cytokine markers in FVB and MMTV mice.** Plasma from a subset of FVB (n=6 normal and n=5 SAD2) and MMTV (n=10 normal and n=10 SAD2) mice were collected, and the cytokine/chemokine 32-plex discovery plex from EVE technologies was used to evaluate changes in cytokines and chemokines in (A) FVB and (B) MMTV mice. The values are displayed as a heatmap showing the medians.

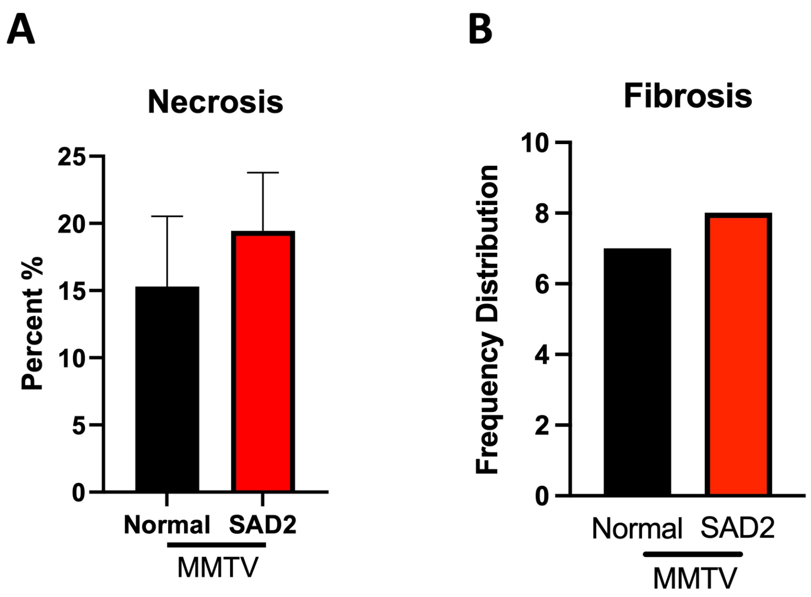


**Suppl. Fig. 6. Necrosis and fibrosis scoring in MMTV tumors.** The tumor tissues from the MMTV mice (n=9 normal chow and n=9 SAD2) were graded for necrosis and fibrosis. (A) The percentage of necrosis was estimated for each tumor sample. (B) Fibrosis was graded based on the presence of fibrosis in the tumor. The percentage of necrotic cells was plotted as the mean ± SEM, and the presence of fibrosis was plotted as a frequency distribution via GraphPad Prism.

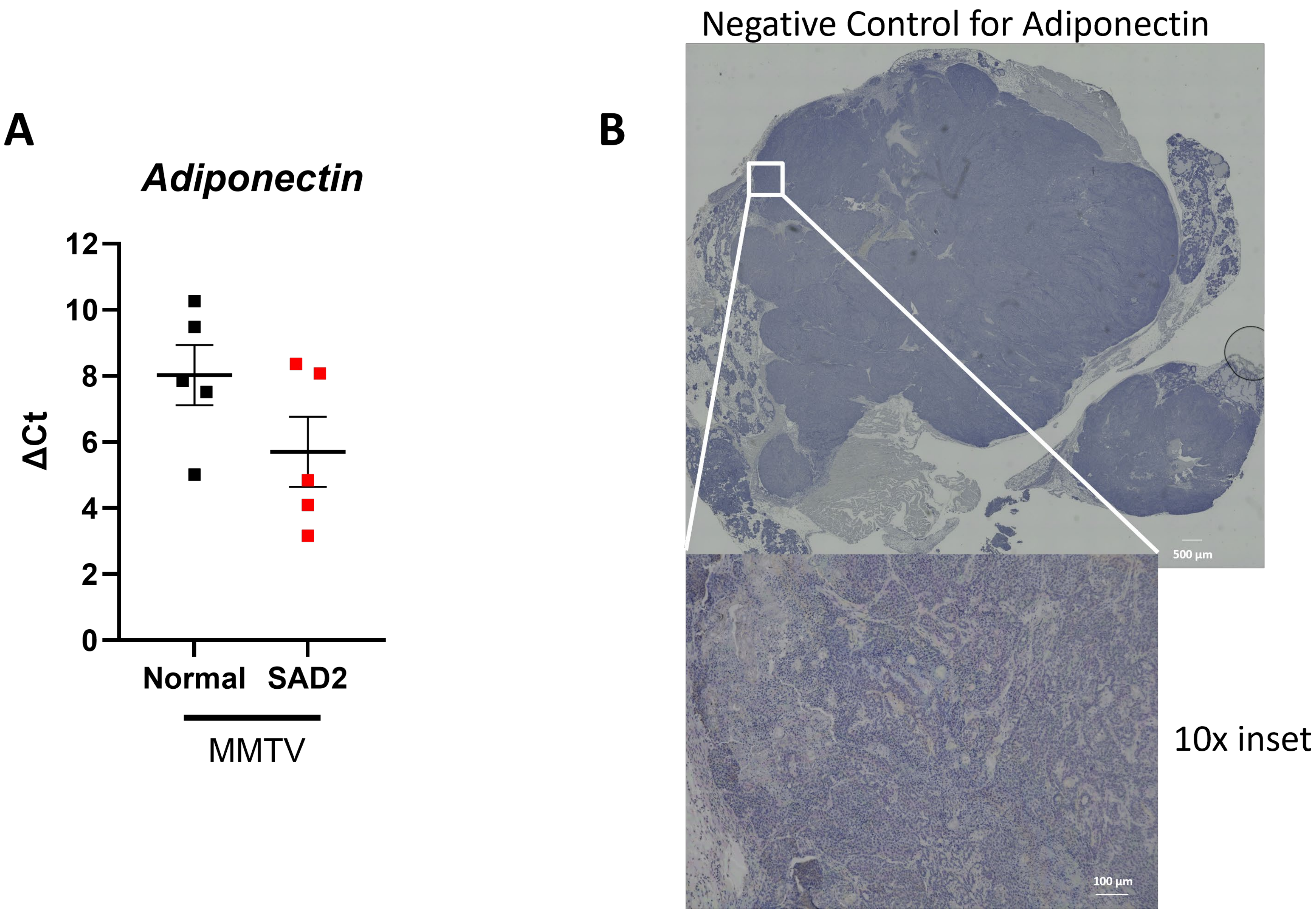


**Suppl. Fig. 7. Adiponectin expression in tumors from MMTV-treated mice. A.** Adiponectin gene expression in a subset of MMTV mouse tumors (n= 9 each group) was measured using the CFX Opus 96 Real-Time PCR System in a subset of MMTV mice tumors. ∆Ct values were calculated and are displayed as the means ± SEMs via GraphPad Prism. **B.** Negative isotype Ig control slide for grading adiponectin staining.

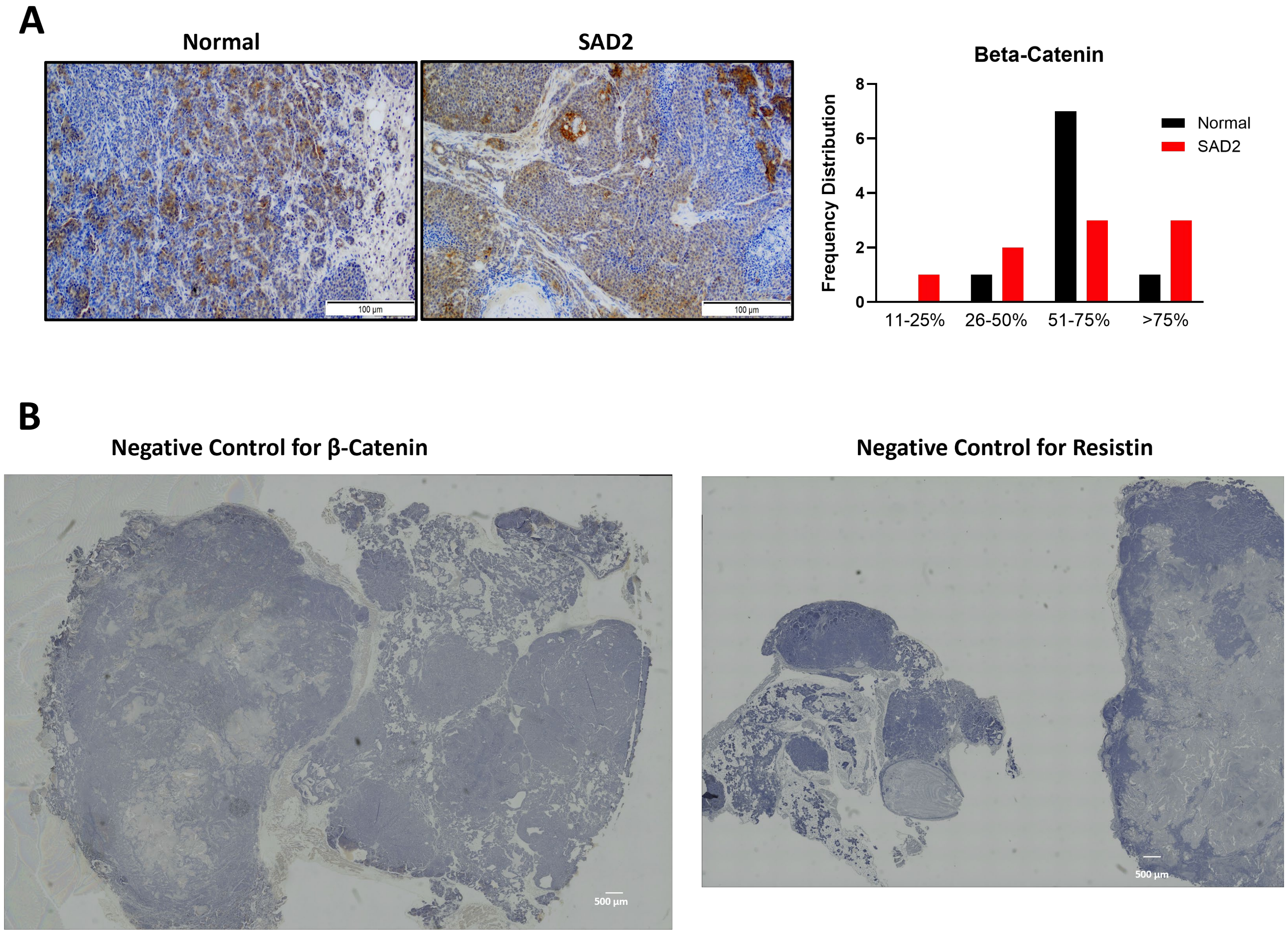


**Suppl. Fig. 8. β-Catenin levels in MMTV mice. A.** Immunohistochemistry for β-catenin was performed on a subset of tumors from individual mice (n=9 normal chow and n=9 SAD2). The levels were graded on the basis of the staining intensity in the tumors. The values were graphed via GraphPad Prism and are presented as the means ± SEMs. **B.** Negative isotype Ig control slide for grading β-catenin and resistin.

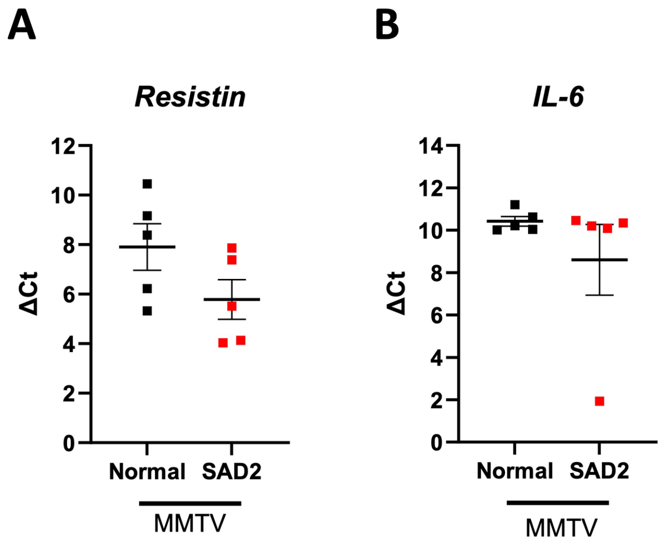


**Suppl. Fig. 9. Gene expression levels of resistin and IL-6 in MMTV mice.** The gene expression of resistin and interleukin 6 (IL-6) in subsets of MMTV mouse tumors (n=5 for each group) was evaluated via the CFX Opus 96 Real-Time PCR System. (A) Resistin and (B) IL-6 ΔCt values were calculated and are presented as the means ± SEMs via GraphPad Prism.

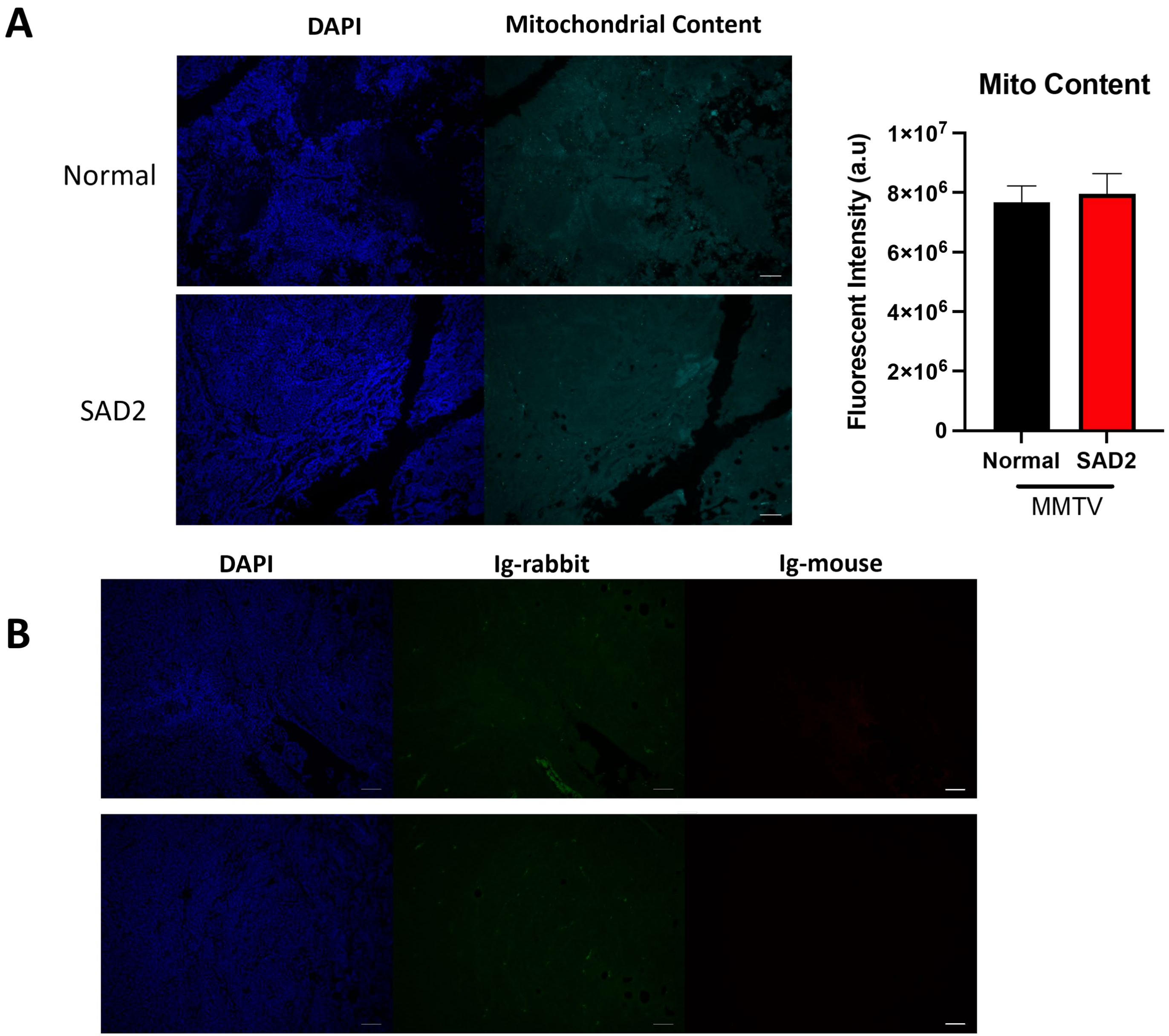


**Suppl. Fig. 10. Mitochondrial content in MMTV mice.** Immunofluorescence staining for total mitochondrial content was performed in a subset of tumors from MMTV mice (n=8 normal chow and n=10 SAD2). A binary threshold was used to measure the fluorescence intensity. The image is representative, and the scale is 100 μm. GraphPad Prism was used to plot the means ± SEMs. **B.** Isotype Ig antibody staining control with 546 and 647 secondary antibody. The scale is 100 μm.

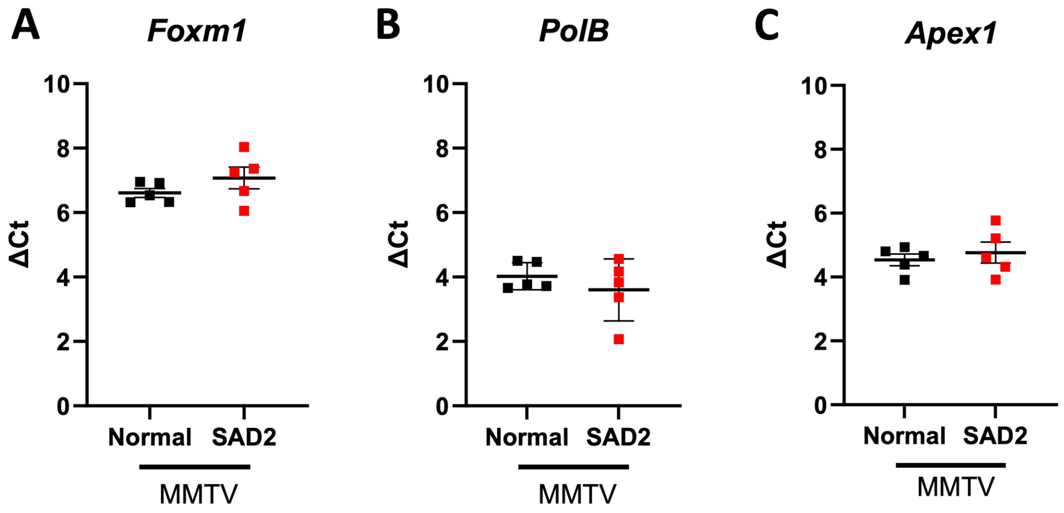


**Suppl. Fig. 11. Gene expression of DNA repair markers in MMTV mouse tumors.** DNA damage and response markers: (A) Forkhead box M1 (*Foxm1*), (B) DNA polymerase beta (*PolB*), and (C) apurinic/apyrimidinic endonuclease 1 (*Apex1*) gene expression in a subset of MMTV mouse tumors (n= 5 each group) was measured via the CFX Opus 96 Real-Time PCR System. The values were calculated as the ΔCt and are displayed in GraphPad Prism as the mean ± SEM.
